# LTF Induces Radioresistance by Promoting Autophagy and Forms an AMPK/SP2/NEAT1/miR-214-5p Feedback Loop in Lung Squamous Cell Carcinoma

**DOI:** 10.7150/ijbs.78669

**Published:** 2023-02-27

**Authors:** Junmiao Wen, Wang Zheng, Liang Zeng, Boyan Wang, Donglai Chen, Yongbing Chen, Xueguan Lu, Chunlin Shao, Jiayan Chen, Min Fan

**Affiliations:** 1 Department of Radiation Oncology, Fudan University Shanghai Cancer Center, Shanghai 200032, China.; 2 Department of Oncology, Shanghai Medical College, Fudan University, Shanghai 200032, China.; 3 Institute of Thoracic Oncology, Fudan University, Shanghai 200032, China.; 4 Institute of Radiation Medicine, Shanghai Medical College, Fudan University, Shanghai 200032, China.; 5 Department of Radiation Oncology, The First Affiliated Hospital of Nanjing Medical College, Nanjing 210029, China.; 6 Department of Thoracic Surgery, Zhongshan Hospital, Fudan University, Shanghai 200032, China.; 7 Department of Thoracic Surgery, the Second Affiliated Hospital of Soochow University, Suzhou 215000, China.

**Keywords:** lung cancer, radioresistance, LTF, autophagy

## Abstract

Radiotherapy is the most predominant treatment strategy for lung squamous cell carcinoma (LUSC) patients, but radioresistance is the major obstacle to therapy effectiveness. The mechanisms and regulators of LUSC radioresistance remain unclear. Here, lactotransferrin (LTF) is found to be significantly upregulated in radioresistant LUSC cell lines (H226R and H1703R) and clinical samples and promotes radioresistance of LUSC both *in vitro* and *in vivo*. Comprehensive enrichment analyses suggested that LTF potentially modulates autophagy in LUSC. Interestingly, the level of autophagy was raised in the radioresistant cells, and suppression of autophagy sensitized LUSC to irradiation. Functional experiments showed that LTF deficiency inhibits cellular autophagy through the AMPK pathway, ultimately leading to radiosensitization. Mechanistically, LTF can directly interact with AMPK to facilitate its phosphorylation and activate autophagy signaling. Moreover, NEAT1 functions as a ceRNA that targets miR-214-5p resulting in an increased LTF expression. Intriguingly, SP2, a transcription factor regulated by AMPK, induced NEAT1 expression by directly binding to its promoter region and thus forming a LTF/AMPK/SP2/NEAT1/miR-214-5p feedback loop. Our work reveals for the first time that LTF induces radioresistance by promoting autophagy and enhancing its self-expression via forming a positive feedback loop, suggesting that LTF is an appealing radiosensitization target for treating LUSC.

## Introduction

Nowadays, lung cancer remains the leading cause of cancer-related death in the world, which accounts for 21% of all cancer mortality 1. Non-small cell lung cancer (NSCLC), the most common subtype of lung cancer, is comprised primarily of two histopathological types: adenocarcinoma (LUAD) and squamous cell carcinoma (LUSC) 2. Due to the rapid progression of the disease, most LUSC patients are initially diagnosed with a late stage and thus have a worse prognosis than LUAD 3. Despite the recent advancements in the treatment of LUSC, including immune checkpoint inhibitor therapy, the overall 5-year survival rate of these patients is still around 20% 4. Radiation therapy is one of the most common methods of lung cancer management, intrinsic and acquired radioresistance is the main obstacle that causes limited therapeutic efficacy in LUSC patients 5. Thus, overcoming radioresistance could improve the therapeutic outcomes of patients with LUSC.

Autophagy, a common phenomenon that could be induced by radiotherapy in tumor cells, plays a key role in the regulation of radioresistance. Recently, researchers have found that regulation of autophagy contributes to either radiosensitization or radioresistization under different conditions. Our previous studies also suggested that the inhibition of autophagy enhances radiosensitivity in various tumors 6-8. However, the relationships between autophagy and radioresistance of LUSC have rarely been reported. Thus, it is of great importance to disclose the molecular mechanisms of autophagy and LUSC radioresistance.

LTF (lactotransferrin), which we know about, is an iron-binding protein that was initially discovered in mammary secretions 9,10. Previous studies indicated that LTF has many important biological functions and possesses antiviral, anti-inflammatory, and immune regulatory activities 11,12. It has been suggested in some reports that the LTF can regulate cell growth, migration, and invasion in several cancers 13. For example, previous studies have found that LTF is associated with proliferation, migration, invasion and promoted apoptosis of clear cell renal cell carcinoma 14,15. LTF has also been suggested to downregulate the development of nasopharyngeal carcinoma by inhibiting the proliferation through induction of cell cycle arrest and modulation of the MAPK signaling pathway 16. However, recent studies have also demonstrated a positive correlation between LTF expression and radioresistance of nasopharyngeal carcinoma 17. Thus, LTF appears to exhibit different expression patterns and functions in different types of tumors, which garnered our interest. Moreover, to our knowledge, the functional roles of LTF in regulating radioresistance of LUSC have not been reported.

In the current study, we found that LTF is significantly upregulated in LUSC radioresistant cells and clinical samples and associated with an unfavorable prognosis. Furthermore, we explored the molecular mechanism and found that LTF directly interacts with AMPK to facilitate its phosphorylation and promotes radioresistance of LUSC by activating autophagy through the AMPK/SP2/NEAT1/miR-214-5p feedback loop. Altogether, our research elucidated a new function of LTF in modulating autophagy and suggested that LTF may serve as a target for radiotherapy sensitization in LUSC.

## Materials and Methods

### Cell culture and irradiation

Human LUSC cell lines (H226 and H1703) were purchased from the Shanghai Institute of Biochemistry and Cell Biology (Shanghai, China). Cells were maintained in RPMI-1640 medium (Gibco) supplemented with 10% FBS (Gibco), authenticated by Short Tandem Repeat (STR) assay, and cultured in a 37 °C incubator with 5% CO_2_.

To generate radioresistant cell lines, H226 and H1703 cells were irradiated with fractionated doses of 2 Gy (60 Gy in total) of X-rays (X‐RAD 320, PXI Inc., North Branford, CT; 12 mA, 2 mm aluminum filtration) at a dose rate of 0.883 Gy/min. After each irradiation, the cells were passaged two or more times so that they had enough vitality for the next irradiation. After fractionated irradiation of 60 Gy, the surviving cells, named H226R and H1703R cells, became more radioresistant than the parental cells. Parental cells underwent the same treatment as radioresistant cells, except irradiation.

### Clonogenic survival assay

Specific steps of the clonogenic assay procedure have been described previously 7,8.

### Cell invasion and migration assays

Transwell assays (Sigma Aldrich, USA) were used to measure cell invasion and migration as described previously 8.

### Tandem mass tag (TMT) quantitative proteomic analysis

Proteins were extracted from the aforementioned LUSC radioresistant cell lines and the parental cells. Total protein concentrations were estimated with the bicinchoninic acid assay (BCA Protein Assay Kit; Beyotime Biotechnology). A quantity of 0.2 mg of protein from each sample was used for TMT analysis following the manufacturer's protocol (Genechem, Shanghai, China). Specific steps have been described previously 7,18.

### RNA isolation and quantitative real-time PCR (qRT-PCR)

Total RNA was extracted from LUSC cells for RT-PCR using total RNA Kit I (Omega, Norcross, GA, United States). Reverse transcription of total RNA to cDNA was carried out in 20 µL reaction reagents of the qRT-PCR Kit (Tiangen, Beijing, China) according to the manufacturer's protocol. Specific primers were listed in ***Table S1***. The optimal PCR amplification procedure included: pre-denaturation at 95 °C for 15 minutes, denaturation at 95 °C for 10 seconds, and annealing and extension at 60℃ for 32 seconds for 40 cycles.

### Transient transfection

The overexpression LTF and lncRNA NEAT1 were produced with pcDNA3.1 (Invitrogen™, USA) and the oe-NC was pcDNA 3.1 vector with inserted scrambled sequences. Meanwhile, two specific siRNAs of LTF and siRNA of lncRNA NEAT1 were designed and offered by GenePharma (Shanghai, China). Based on different sequences, siRNAs were named siLTF-1, siLTF-2, and siNC. Moreover, miR-214-5p mimics, miR-214-5p inhibitors, and the related negative control (NC) were purchased from GenePharma (Shanghai, China). The siRNA constructs were listed in ***Table S2***. Cells were transfected using Lipofectamine 2000 reagent (Invitrogen, Carlsbad, CA, USA) according to the instructions, and the effect of transfection was evaluated by qRT-PCR.

### Western blotting assay

The procedure followed our previous research 7,18, and the detailed information of antibodies was listed in ***Table S3***.

### Co-immunoprecipitation (Co-IP) assay

In brief, the protein was extracted in RIPA lysis buffer with protein inhibitor on ice and then centrifuged at 12,000 g for 10 min. The protein A/G agarose beads were incubated with antibodies and protein samples overnight at 4 °C with rotation. After 12 h, the complexes were washed and then obtained for Western blotting analysis. Information on the antibodies is listed in ***Table S3***.

### Transmission electron microscopy

For ultrastructural analysis of autophagosomes, transmission electron microscopy (TEM) was carried out. H226R and H1703R control cells or LTF knockdown cells were fixed in ice-cold 2.5% glutaraldehyde at 4 °C overnight and post-fixed in 1% osmium tetroxide (OsO4) at 4 °C for 2 h. The samples were subsequently dehydrated through an ethanol solution from 50 to 100% and embedded in Epon812 epoxyresin. Ultrathin sections were then collected and stained with either uranyl acetate or lead citrate and reviewed under a TEM (JEM-2000EX, JEOL, Japan).

### Autophagy flux assay

Cells were plated at a density of 2 × 10^5^ per well and allowed to adhere overnight. Cells in about 70% confluence were transfected with mRFP-GFP-LC3 double-labeled adenovirus (Ad-mRFP-GFP-LC3) to label autophagosome (Hanbio Biotechnology Co., Shanghai, China) according to the manufacturer's instruction. After 2 h of transfection, the cells were cultured in a fresh medium for 48 h then washed with pre-cooled PBS twice and stained with DAPI. The intracellular autophagy was observed by a high-content imaging system (ImageXpress Micro 4, Molecular Devices, San Francisco, CA, United States). Double labeling of LC3 (green) and mRFP (red) immunofluorescence corresponds to changes in autophagic flux. When autophagy and lysosome fusion occurs, LC3-GFP fluorescence is quenched, and only red fluorescence can be detected. After merging the red and green fluorescence images, yellow spots in the cell image symbolize autophagosomes.

### Dual immunofluorescence assay of LTF and AMPK

Cells were fixed in 4% formaldehyde followed by permeabilization treatment with 0.5% Triton X-100 for 10 min, blocked with 0.1% PBS-Tween solution for 1 h, and probed with rabbit-anti-LTF antibody for 12 h and mouse-anti-AMPK antibody (Abcam) for another 12 h, followed by incubation with Alexa Fluor 555 goat anti-rabbit antibody for 1 h and Alexa Fluor 488 goat anti-mouse antibody for another 1 h. After washing with pre-cold PBS triply, cell nuclei were stained with DAPI (Invitrogen, CA, USA) at a concentration of 1.43 µM for 10 min.

### Chromatin immunoprecipitation

Chromatin immunoprecipitation (ChIP) was performed according to the protocol of Magna ChIP TM A/G kit (Millipore, USA). In brief, LUSC cells were fixed in 1% formaldehyde for 10 min at room temperature and then cracked by cell lysis buffer for 15 min on ice, followed by sonication to shear DNA into fragments between 200 and 700 base pairs. Appropriate antibodies and protein A/G beads were used to incubate with DNA fragments as described. Normal rabbit or mouse IgG was used as the negative control. After washing with low salt, high salt, LiCl buffer, and TE buffer, the elution buffer was used to harvest the chromatin fragments. NEAT1 enrichment degree was examined by quantitative PCR.

### *In vivo* tumor growth and irradiation

The *in vivo* experiments were approved by the Institutional Animal Care and Use Committee of Fudan University and abided by Institutional Guidelines and Protocols (approval number FUSCC-IACUC-S2022-0059). Specific steps have been described previously 6. In brief, five-week-old male BALB/c nude mice (18-20 g) were purchased from SIPPR/BK Lab. Animal Co. Ltd. (Shanghai, China) and maintained in a stable environment (23 ℃, 12 h dark and 12 h light) for one week before experiments. For establishing the tumor xenograft model, 5 × 10^6^ H226R or H1703R cells with or without siLTF interference were implanted subcutaneously into the lateral aspect of the rear leg. When the xenograft volumes approached approximately 50 mm^3^, cholesterol-modified LTF siRNA or its control (RiboBio, Guangzhou, China) was administrated intratumorally every 3 days until the mice were sacrificed. When the tumor approached 100 mm^3^ approximately, mice were randomized into the IR group and non-IR control group (n = 5 for each group). Mice in the IR group were given a total dose of 24 Gy X-rays irradiation on three consecutive days (8 Gy/day). The perpendicular diameter of each tumor was measured every three days with digital calipers, and the tumor volume was calculated using a formula (L × W^2^) × π/6, where L and W are the tumor's length and width.

### Patients samples and immunohistochemical staining (IHC)

A total of 110 LUSC patients who underwent radiotherapy were included and divided into radiosensitive and radioresistant groups. The patients' clinicopathological features are summarized in ***Table S4***. The study was approved by the Ethics Committee of the Fudan University Shanghai Cancer Center. All patients provided written informed consent. Formalin-fixed and paraffin-embedded tissue sections from human LUSC and xenograft tumor tissues were first deparaffinized in xylene and subsequently stained with antibodies targeting LTF. The expression of LTF in clinical specimens was scored semi-quantitatively by two independent pathologists blinded to the clinical data. The IHC score was determined by assigning values based on the percentage of positively stained tumor cells (range from 0 to 100) and the staining intensity (range from 0 to 3); these values were then multiplied to generate an H-score.

### Statistical analysis

The data were analyzed using R software version 3.5.2 (Institute for Statistics and Mathematics, Vienna, Austria; https://www.r-project.org/) or GraphPad Prism 8.0 (GraphPad Prism, California, USA), and were presented as means ± SD 19. Student's t-test or ANOVA was used for group comparisons. The log-rank test and Kaplan-Meier survival curves were employed to compare Relapse-free survival and overall survival between different subgroups. A two-sided *p*-value < 0.05 was considered statistically significant.

## Results

### LTF is upregulated in radioresistant LUSC cells and related to poor prognosis

**Figure 1:**
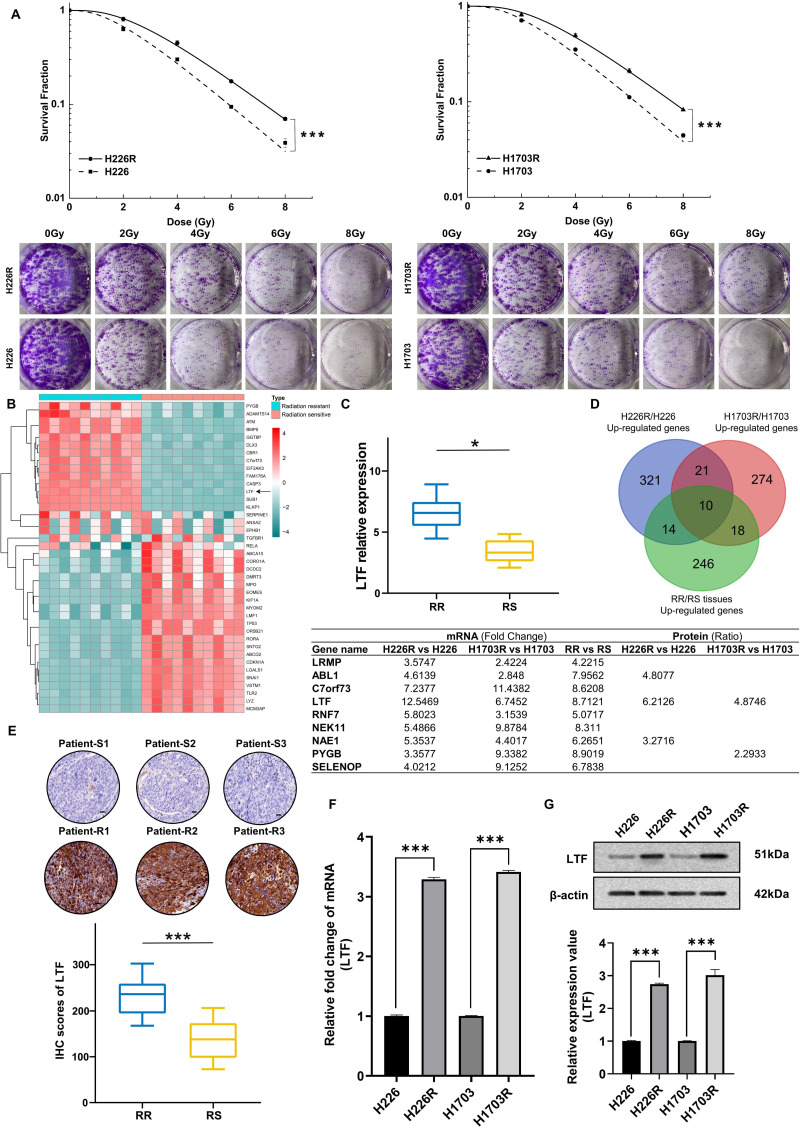
** LTF expression is upregulated and correlated with radioresistance in LUSC tissues and LUSC cell lines. (A)** Clonogenic assays of H226/H226R and H1703/H1703R cells treated with the indicated doses. n = 3 wells/group. **(B)** Clustered heatmap of RNA-seq data showing the top differentially expressed (up- and downregulated) genes in 10 LUSC radiation resistant and 10 sensitive tissues. The black arrowhead shows the LTF gene. **(C)** RT-qPCR analysis of LTF expression in 10 LUSC radiation resistant (RR) and 10 sensitive (RS) tissues. * *p* < 0.05. **(D)** Venn diagram showing the overlap between upregulated genes of H226R/H226, H1703R/H1703 cells and the clinical radioresistant/radiosensitive LUSC samples. The intersection contains ten genes, which are listed next the diagram. **(E)** Representative immunohistochemistry staining against LTF in radiosensitive (patient-S1,2,3) and radioresistant (patient-R1,2,3) clinical LUSC samples (40X). Scale bars, 20 μm. **(F)** qRT-PCR analysis of LTF expression in H226/H226R and H1703/H1703R cells. **(G)** Western blot assay of LTF protein in H226/H226R and H1703/H1703R cells.

To elucidate the molecular mechanisms of radioresistance in LUSC, we established two photon radioresistant cell lines (H226R and H1703R) from parental H226 and H1703 cells and further verified their radioresistance by clonogenic assay. As shown in ***Fig. 1A****,* H226R and H1703R cells were significantly more resistant to radiation compared to H226 (*p* = 0.0012) and H1703 cells (*p* = 0.0024), respectively.

To determine the potential genes involved in the radioresistance of LUSC cells, the RNA sequencing (RNA-seq) along with tandem mass tag (TMT) quantitative proteomic analysis was conducted between these two cell lines. The RNA-seq data disclosed that LTF was one of the overlapping genes within the differentially regulated genes from the H226R/H226 and H1703R/H1703 datasets (***Fig. S1A and S1B***). As shown in ***Fig. S1C*** and ***S1D***, the TMT quantitative proteomic analysis also indicated significant upregulation of LTF protein both in H226R/H226 cells (ratio = 6.213, *p* < 0.05) and H1703R/H1703 cells (ratio = 4.875, *p* < 0.05). Additionally, RNA-seq and qRT-PCR analysis of clinical patient samples also revealed higher LTF expression in radioresistant than radiosensitive tissues (***Fig. 1B, C***). LTF was one of the ten genes that were identified in the intersection of the upregulated genes from the aforementioned three RNA-seq datasets (***Fig. 1D***). Further analysis of the TCGA database of 435 patients revealed that LTF is overexpression and correlated with poor prognosis in LUSC (***Fig. S1E, F***). In addition, for patients underwent RT in FUSCC, OS and RFS of those with high LTF expression were worse than in patients with low LTF expression (***Fig. S1G***). Multivariate Cox regression analysis showed that LUSC patients with higher LTF expression had a significantly worse prognosis (***Fig. S1H***). We further performed IHC staining against LTF in a set of radiosensitive and radioresistant clinical LUSC specimens, and the results indicated higher expression in the radioresistant group than in the radiosensitive group (***Fig. 1E***).

Next, we validated the expression of LTF in radioresistant cells by conducting qRT-PCR and WB. It was observed that the expression levels of LTF mRNA and protein in H226R and H1703R were significantly upregulated compared to their parental cells (***Fig. 1F, G***). These results imply that LTF overexpression is involved in the development of radioresistance and may predict a poor prognosis of LUSC.

### Inhibition of LTF enhanced radiosensitivity of LUSC cells *in vitro* and *in vivo*

**Figure 2:**
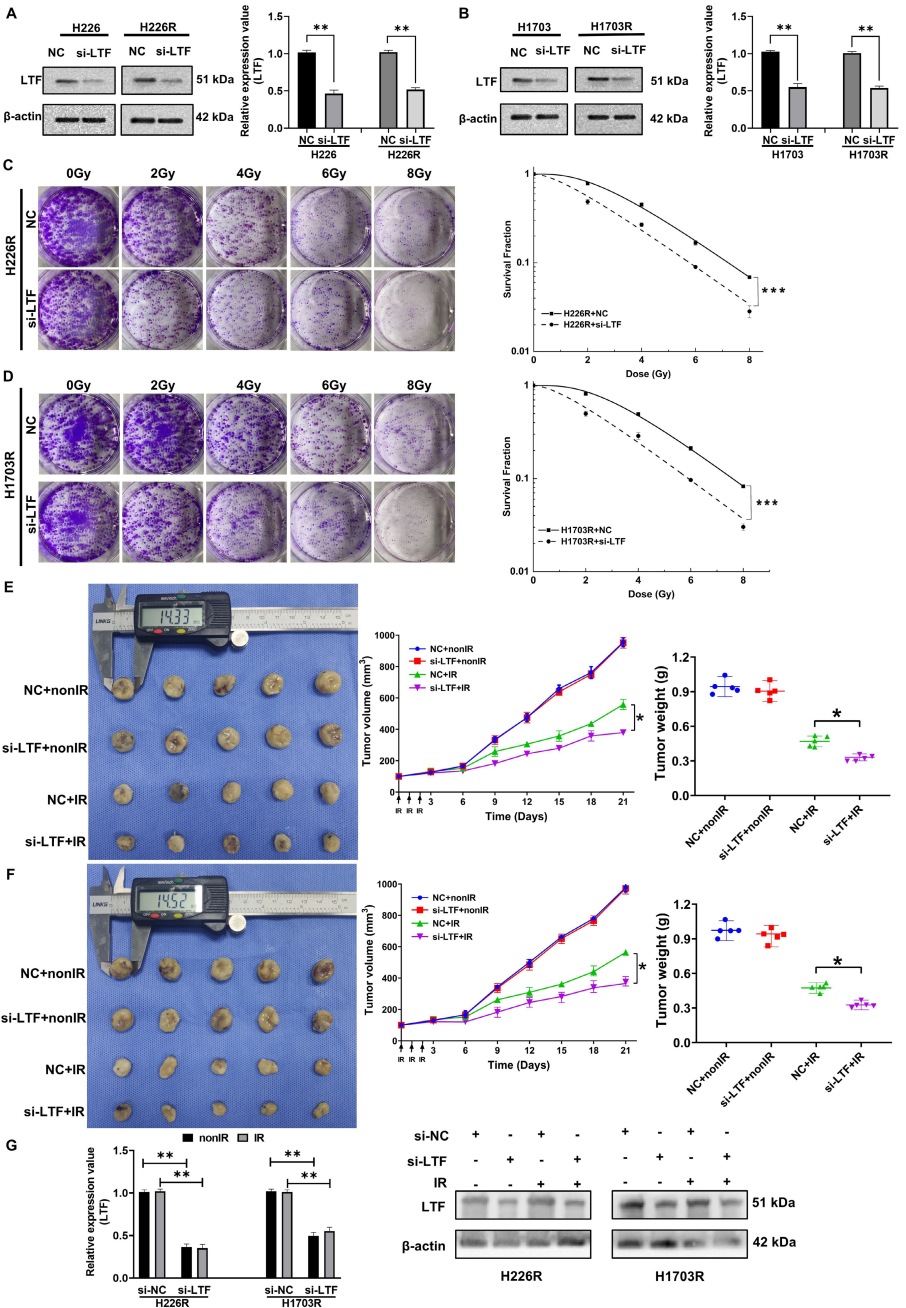
** Inhibition of LTF enhanced radiosensitivity of LUSC cells *in vitro* and *in vivo*. (A)** Western blot analysis of LTF expression in nonirradiated or irradiated H226 and H226R cells following si-LTF transfection. ** *p* < 0.01 between indicated groups, *** *p* < 0.001 between indicated groups. **(B)** Western blot analysis of LTF expression in nonirradiated or irradiated H1703 and H1703R cells following si-LTF transfection. ** *p* < 0.01 between indicated groups, *** *p* < 0.001 between indicated groups.** (C)** Dose responses of survival factions of H226R cells before and after si-LTF transfection. *** *p* < 0.001 between indicated groups. **(D)** Dose responses of survival factions of H1703R cells before and after si-LTF transfection. *** *p* < 0.001 between indicated groups. **(E)** Tumor growth curves, tumor weight and representative image of H226R xenograft tumors under different treatments of si-NC, si-LTF, si-NC + ionizing radiation (IR), and si-LTF + IR, respectively. * *p* < 0.05 between same cells before and after irradiation. Tumor volume was measured every three days with a digital caliper and calculated using the formula (L ×W^2^) × π/6. Each bar represents the mean ± SD derived from five independent experiments. **(F)** Tumor growth curves, tumor weight and representative image of H1703R xenograft tumors under different treatments of si-NC, si-LTF, si-NC + ionizing radiation (IR), and si-LTF + IR, respectively. * *p* < 0.05 between same cells before and after irradiation. Tumor volume was measured every three days with a digital caliper and calculated using the formula (L ×W^2^) × π/6. Each bar represents the mean ± SD derived from five independent experiments. **(G)** Western blot of LTF expression level in dissected tumors above. ** *p* <0.01 between indicated groups, *** *p* < 0.001 between indicated groups.

To further assess the potential involvement of LTF in the radiosensitivity of LUSC, siRNA targeting LTF (si-LTF) and a scramble control siRNA (si-NC) were transferred into LUSC cells. The expression of LTF in H226R, H1703R and their parental cells was effectively silenced by si-LTF (***Fig. 2A, B***). It was found that transfection of cells with si-LTF significantly sensitized H226R and H1703R cells to irradiation and reduced cell survival (***Fig. 2C, D***). Consistently, the effective transfection of si-LTF remarkably reduced the survival fractions of H226 and H1703 cells after irradiation (***Fig. S2A, B***). Therefore, transfection of si-LTF markedly sensitized LUSC cells to irradiation. Moreover, we wondered whether LTF regulated the migration and invasion ability of LUSC cells. According to the transwell assay results, downregulation of LTF remarkably suppressed the migration and invasion of LUSC cells (***Fig. S2C, D***).

To determine the contribution of LTF in the tumor radioresistance *in vivo*, we administrated cholesterol-modified si-LTF or its control into the xenograft of H226R and H1703R cells every 3 days during tumor growth. H226R and H1703R cells transfected with si-NC and si-LTF were subcutaneously injected into athymic nude mice. When the tumor approached about 100 mm^3^, they were locally irradiated with fractional doses of 8 Gy/day in three consecutive days and then the growth curves of xenografts were observed. As expected, the intra-tumoral interference of LTF further enhanced radiation-induced growth suppression of LUSC xenografts of both H226R and H1703R cells (***Fig. 2E and F***). Notably, we found that the expression of LTF in dissected tumors was upregulated after irradiation and significantly downregulated in si-LTF transfected LUSC cells (***Fig. 2G***). Together, these data suggest that LTF is necessary for the radioresistance of LUSC cells *in vitro* and *in vivo*.

### LTF regulated LUSC radioresistance through the autophagy induction

**Figure 3:**
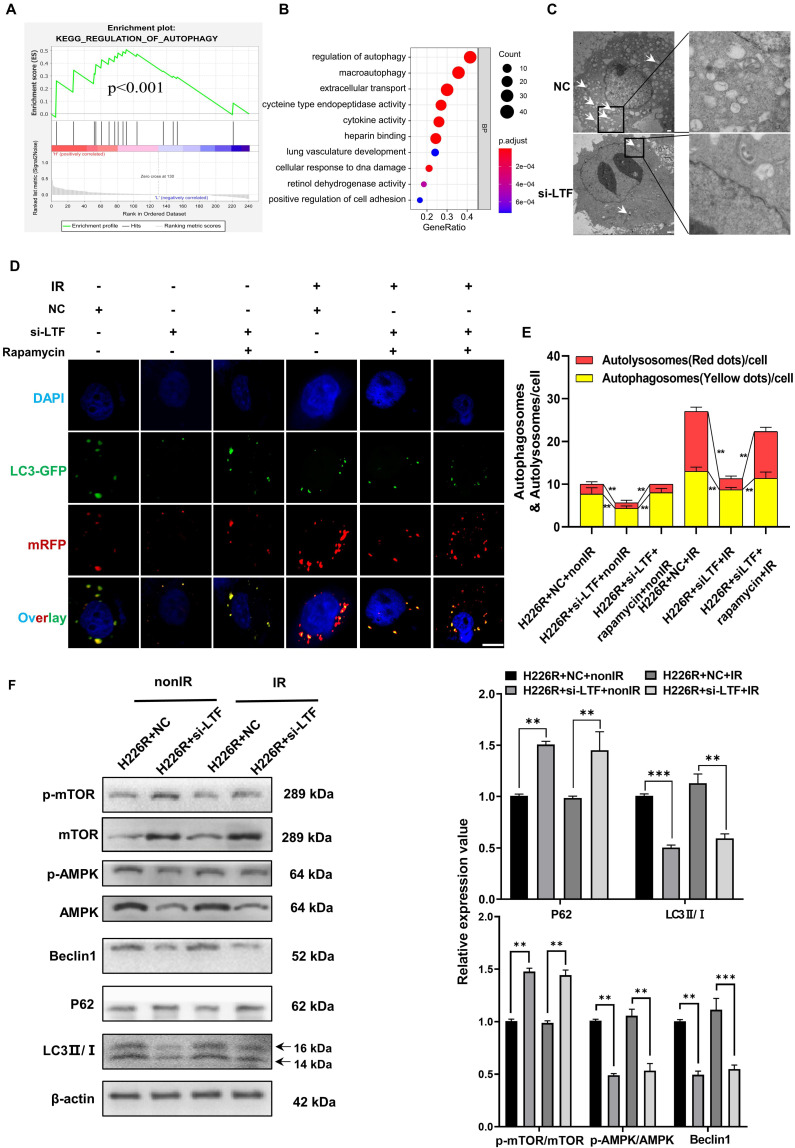
** Knockdown of LTF inhibits the level of autophagy via the AMPK/mTOR/Beclin1 axis in LUSC cells. (A)** GSEA analysis of LTF based on expression in the TCGA-LUSC dataset. NES: normalized enrichment score; NOM p-value: nominal p value; FDR q-val: false discovery rate.** (B)** GO analysis were performed to detect the function of LTF in cell processes in the TCGA-LUSC dataset.** (C)** The autophagosomes of H226R cells with control or stable knockdown of LTF were examined with transmission electron microscopy (TEM). Autophagosomes was indicated by white arrows. Scale bar = 5 μm (left) or Scale bar = 1 μm (right). **(D, E)** Fluorescence images of H226R cells transfected with si-LTF and mRFP-GFP-LC3-tagged adenovirus (×40) after 6 Gy irradiation or not. Red dots indicate autolysosomes while yellow dots indicate autophagosomes in overlays. Nuclei were stained with DAPI. Scale bars: 10 μm. The average number of autophagosomes and autolysosomes in each indicated cell was quantified. ** *p* < 0.01. **(F)** Western blot analysis of mTOR, p-mTOR, AMPK, p-AMPK, Beclin1, P62 and LC3 expression in nonirradiated or irradiated H226R cell following si-LTF transfection. ** *p* < 0.01 between indicated groups, *** *p* < 0.001 between indicated groups.

Our previous research provided evidence that autophagy was involved in the radioresistance of tumor cells 6,8. Intriguingly, Gene set enrichment analysis (GSEA) indicated that LTF was positively related to autophagy pathways (***Fig. 3A***). GO analysis also suggested that LTF may affect the process of autophagy in LUSC (***Fig. 3B***). Therefore, we transfected LUSC cells with Ad-mRFP-GFP-LC3 to label autophagosomes to determine whether autophagy is involved in the radioresistance of LUSC cells. As expected, in comparison with H226 and H1703, radioresistant cells H226R and H1703R exhibited higher intrinsic autophagy level (***Fig. S3A***). Western blot assay also indicated that the ratio of LC3II/LC3I (an autophagic marker) increased while the autophagy substrate p62 decreased in H226/H226R and H1703/H1703R cells (***Fig. S3B***). Furthermore, silencing LC3 by LC3 siRNA significantly decreased the survival and the autophagosome formation of radioresistant LUSC cells (***Fig. S3C-E***). These results demonstrated that autophagy promoted the radioresistance of LUSC cells.

Moreover, The TEM data also revealed that autophagy levels were decreased in LTF knockdown LUSC radioresistant cells compared to control cells (***Fig. 3C and Fig. S4A***). For sake of investigating the role of LTF in autophagy-regulated radioresistance of LUSC cells, H226R and H1703R cells were transfected with si-LTF and Ad-mRFP-GFP-LC3. As shown in ***Fig. 3D, E*** and ***Fig. S4B***, exposure to 6Gy X-rays significantly enhanced the autophagic flux of LC3 while the number of autophagic LC3 spots in the si-LTF transfected cells decreased sharply in comparison with the si-NC group. In addition, rapamycin, a specific inducer of autophagy, could reverse si-LTF-mediated inhibition of autophagy in radioresistant LUSC cells. This phenomenon was further verified by the increased p62 expression and the decreased LC3 II/I ratio in the si-LTF transfected LUSC cells (***Fig. 3F, Fig. S4C***).

### LTF promoted autophagy by interacting with AMPK

Next, we determined the efficiencies of various autophagy-related pathways in cells in which the expression of LTF was manipulated. Linear correlation analyses indicated a correlation between LTF and AMPK/mTOR/Beclin1 in LUSC tissues collected from FUSCC (***Fig. S4D-H***). Since AMPK, mTOR and Beclin1 are all well-known regulators of autophagy, we analyzed the relationship of LTF with the AMPK/mTOR/Beclin1 signaling pathway. As shown in ***Fig. 3F*** and ***Fig. S4C***, silencing of LTF led to a significant upregulation of p-mTOR and degradation of p-AMPK, Beclin1 both in H226R and H1703R cells.

To reveal the regulatory mechanisms of LTF in the AMPK/mTOR pathway, we performed immunofluorescence and Co-IP assays to detect the interaction of LTF with AMPK. The results disclosed that LTF and p-AMPK were co-located in the LUSC cells and more LTF and p-AMPK fluorescence puncta were observed in the radioresistant cells than the parental cells.

**Figure 4:**
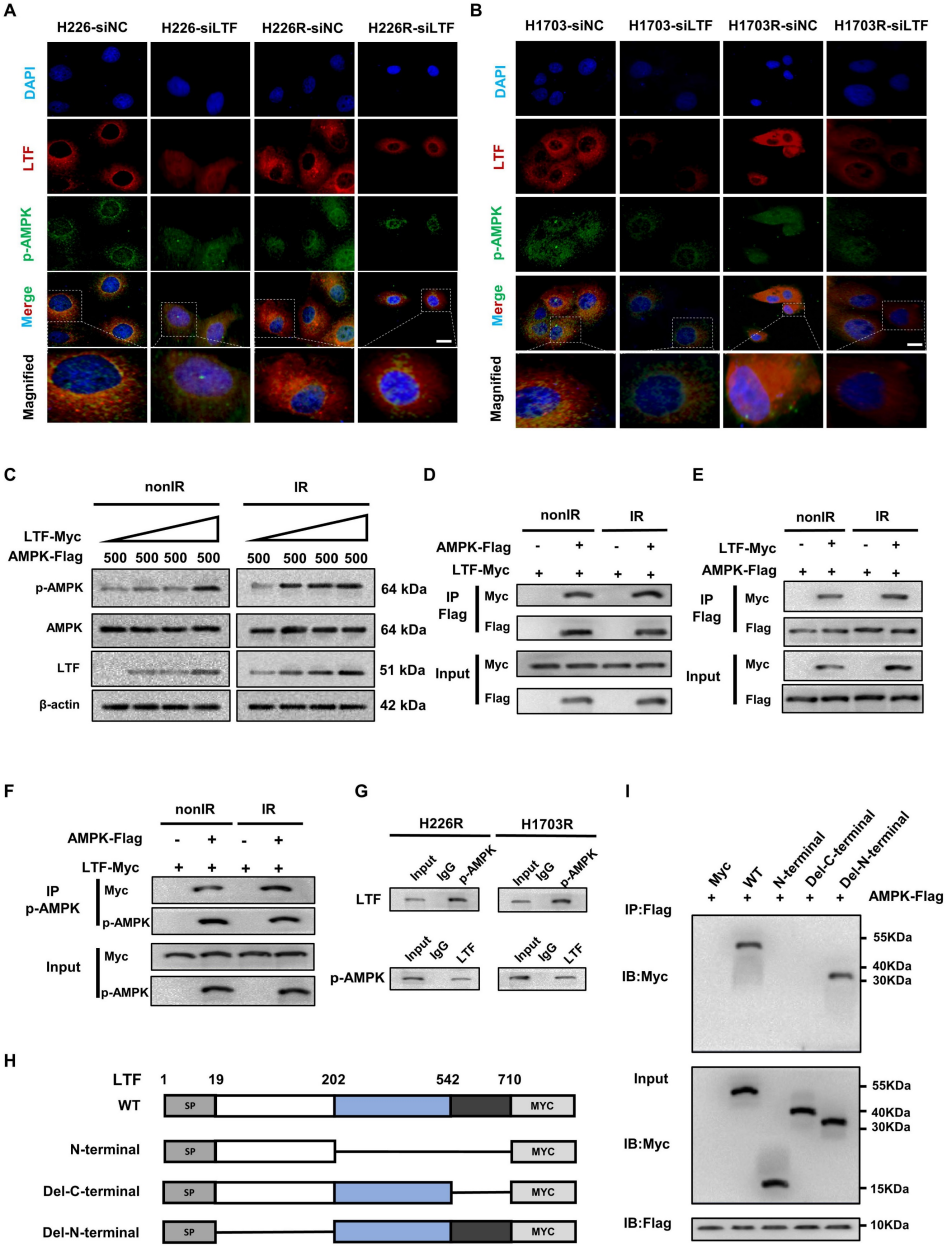
** LTF co-localizes and interacts directly with the C-terminal domain of AMPK to promotes its phosphorylation in LUSC cells. (A)** Representative immunofluorescence images of LTF expression and its distribution in H226-siNC, H226-siLTF, H226R-siNC and H226R-siLTF cells. Scale bars, 20 μm. Relative average optical density (RAOD) of the expression of LTF, p-AMPK proteins and their merged regions in three independent microscopic fields of above LUSC cells. * *p* < 0.05 between indicated groups. **(B)** Representative immunofluorescence images of LTF expression and its distribution in H1703-siNC, H1703-siLTF, H1703R-siNC and H1703R-siLTF cells. Scale bars, 20 μm. Relative average optical density (RAOD) of the expression of LTF, p-AMPK proteins and their merged regions in three independent microscopic fields of above LUSC cells. **(C)** 293T cells cotransfected with 500 ng AMPK-FLAG and increasing concentrations of LTF- Myc were treated with or without irradiation (6 Gy). Twenty-four hours later, the cells were lysed and subjected to WB assays with the indicated antibodies. **(D)** 293T cells were transiently transfected with MYC-tagged LTF with or without FLAG-tagged AMPK and co-immunoprecipitation assays were performed. **(E)** 293T cells were transiently transfected with FLAG-tagged AMPK with or without MYC-tagged LTF and co-immunoprecipitation assays were performed. **(F)** The interaction between LTF and p-AMPK in 293T cells was tested using IP assays. **(G)** Co-IP assays of endogenous proteins of LTF and p-AMPK in H226R (left) and H1703R (right) cells. **(H, I)** Co-IP and western blot assays using Flag antibody showing the interaction between AMPK and LTF protein in 293T cells transfected with a series truncations of Myc-tagged LTF. *p < 0.05 between indicated groups.

Furthermore, si-LTF transfection concurrently decreased the co-localization of LTF and p-AMPK (***Fig. 4A, B***). In addition, we found that in 293T cells, the p-AMPK protein expression was remarkably enhanced in a dose-dependent manner by co-expression with LTF (***Fig. 4C***). Intriguingly, we found that LTF strongly interacted with AMPK and p-AMPK in 293T cells regardless of irradiation status (***Fig.4D-F***). The protein interactions between LTF and p-AMPK were also substantiated in H226R and H1703R cells by applying LTF antibody or p-AMPK antibody against the endogenous protein (***Fig. 4G***). Moreover, the carboxy-terminal of LTF (amino acids 542-710) was necessary for its binding to AMPK (***Fig. 4H, I***).

Taken together, the above results demonstrate that LTF contributes to the radioresistance of LUSC cells by directly interacting with AMPK, promoting AMPK phosphorylation and inducing autophagy.

### miR-214-5p and LTF were mutually antagonistic

**Figure 5:**
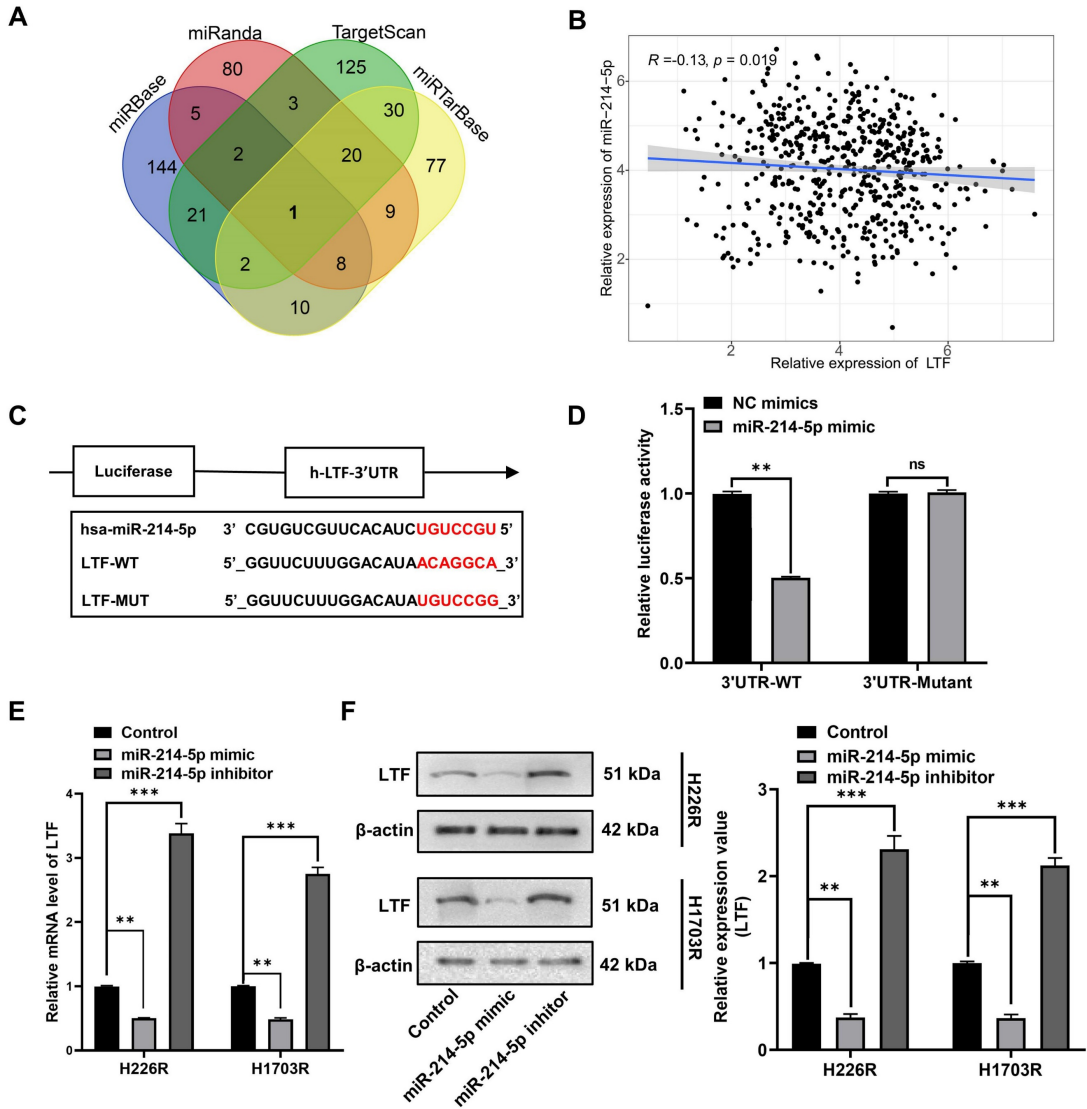
** miR-214-5p directly targeted LTF in LUSC cells. (A)** Venn plot of predicted target miRNAs of LTF. **(B)** The correlation analysis of LTF and miR-214-5p expression. **(C)** The binding site of miR-214-5p and LTF was predicted by the online website TargetScan.** (D)** The luciferase activity of LTF-WT and LTF-MUT following transfection with NC-mimic and miR-214-5p mimic was detected by dual luciferase reporter gene assay. **(E, F)** The mRNA and protein expression of LTF in H226R and H1703R cells following transfection with Control, miR-214-5p mimic and miR-214-5p inhibitor was measured by RT-qPCR and Western blot analysis, respectively.

To determine the upstream regulatory mechanism of LTF, miRNA target prediction databases miRbase, miRanda, miRTarBase and TargetScan were applied to predict the potential miRNA of LTF, which revealed miR-214-5p as the regulator of LTF (***Fig. 5A***). A correlation analysis revealed that the expression of LTF was negatively correlated with the expression of miR-214-5p (***Fig. 5B***). Bioinformatics analysis confirmed the presence of a special binding area between the gene sequence of LTF and miR-214-5p (***Fig. 5C***). Next, a dual-luciferase reporter gene assay revealed that cells co-transfected with LTF-WT and miR-214-5p mimic exhibited reduced luciferase activity, while no significant differences were detected in the luciferase activity of LTF-MUT (***Fig. 5D***). Moreover, it was suggested that the mRNA and protein expression of LTF was notably decreased following miR-214-5p mimic transfection, while an increase was identified by RT-qPCR and Western blot analyses following miR-214-5p inhibitor transfection (***Fig. 5E and F***). These results confirmed that LTF was the target gene of miR-214-5p.

### NEAT1 upregulated LTF mRNA by sponging miR-214-5p

**Figure 6:**
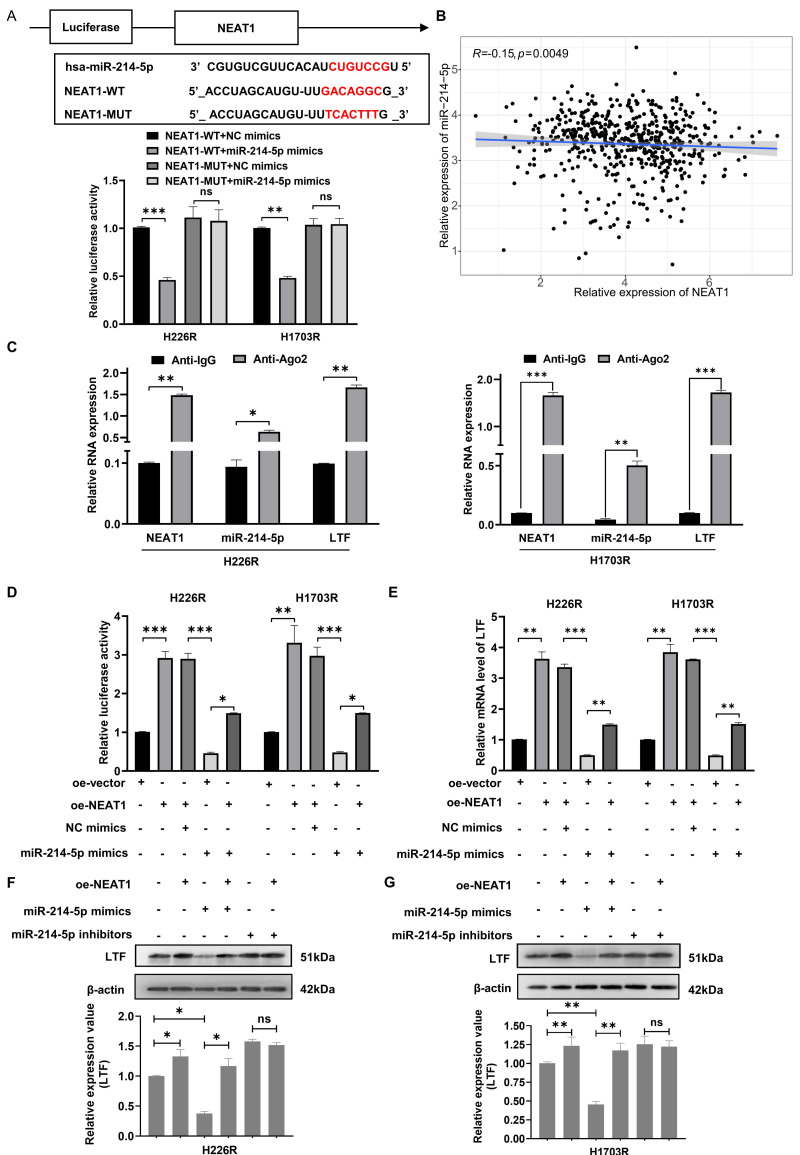
** NEAT1 competitively binds with miR-214-5p to prevent the degradation of its target gene LTF. (A)** The dual-luciferase assay showed that the relative dual-luciferase activity of the NEAT1-WT group was directly inhibited by miR-214-5p mimics. The binding sites were predicted by using the starBasev3.0 database. **(B)** Relationship between NEAT1 expression and miR-214-5p levels in LUSC samples. **(C)** The direct interaction between NEAT1 and miR-214-5p was verified by RIP assay. **(D)** Relative activity of LTF promoter in H226R and H1703R co-transfected with oe-NEAT1 and miR-214-5p mimics in H226R and H1703R cells. **(E)** oe-NEAT1 antagonized the inhibition of miR-214-5p mimics on LTF mRNA expression in H226R and H1703R cells. **(F, G)** Western blot detection of LTF expression levels in H266R (F) and H1703R (G) cells transfected with oe-NEAT1 and cotransfected with miR-214-5p mimics or inhibitors.

Increasing evidence shows that the resistance of anticancer treatments is aggravated through miRNA or mRNA controlled by upstream lncRNA. Thus, we use StarBase 3.0 to screen out the potential ceRNA network shared by LTF and miR-214-5p. The predicted network of ceRNA showed an excellently possible miR-214-5p/LTF axis in LUSC regulated by lncRNA NEAT1. Consequently, we found that the NEAT1 sequence has a possible miR-214-5p binding site, and double luciferase reporter assays confirmed that miR-214-5p indeed binds to NEAT1 (***Fig. 6A***). Moreover, we quantified NEAT1 and miR-214-5p in LUSC samples and found a significantly negative correlation between them (***Fig. 6B***). Anti-Ago2 antibody precipitated NEAT1, miR-214-5p and LTF mRNA indicating that miR-214-5p was involved in RNA-induced silencing complexes (***Fig. 6C***). Besides, qRT-PCR and Western blot assays disclosed that LTF mRNA and protein expressions were lowest in the miR-214-5p mimic group and were rescued following the addition of oe-NEAT1 (***Fig. 6D-G***). These findings support our hypothesis that NEAT1, as a ceRNA of miR-214-5p, regulates the expression of its target gene LTF.

### Involvement of the NEAT1/miR-214-5p/LTF axis in autophagy-mediated radioresistance of LUSC cells

**Figure 7:**
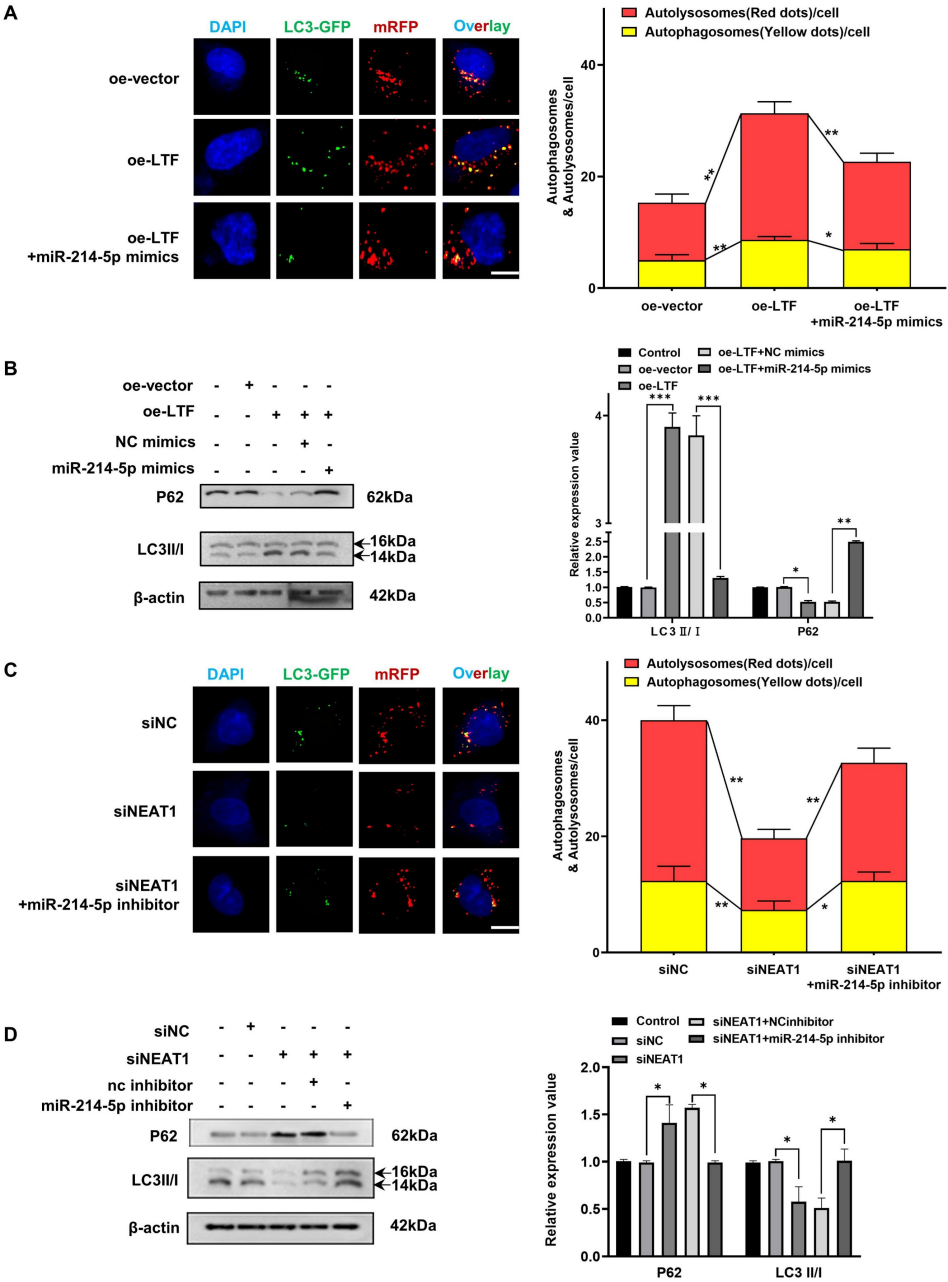
** The NEAT1/miR-214-5p/LTF axis is involved in autophagy-mediated radioresistance of H226 cells.** mRFP-GFP-LC3 **(A)** and Western blot **(B)** assay showed miR-214-5p mimics reversed the effects of oe-LTF on autophagy in H226R cells. Red dots indicate autolysosomes while yellow dots indicate autophagosomes in overlays. Nuclei were stained with DAPI. Scale bars: 10 μm. The average number of autophagosomes and autolysosomes in each indicated cell was quantified. **p < 0.01. **(C)** The effect on H226R cell autophagy levels following transfected with si-NEAT1, si-NEAT1 plus miR-214-5p inhibitor, or the control after X-ray irradiation is tested by autophagic flux analysis. **(D)** Western blots identified the autophagy-related protein expression changes in si-NEAT1 and si-NEAT1 plus miR-214-5p inhibitor transfected H226R cells. β-actin was used as a control.

As we observed that knockdown of LTF increased the radio-sensitivity of LUSC cells by regulating autophagy, we thus aimed to assess whether the NEAT1/miR-214-5p/LTF axis affects the radiosensitivity of LUSC. Clonogenic survival assay demonstrated that transfection of H226R and H1703R cells with oe-LTF increased cell survival capability while miR-214-5p mimics could reverse this effect (***Fig. S5A, B***). Furthermore, it was identified that the number of autophagic LC3 spots and autophagy-related proteins was remarkably increased in the oe-LTF cells, while this positive impression on autophagy could be reversed by the overexpression of miR-214-5p (***Fig. 7A, B and Fig. S6A, B***). These results indicated that miR-214-5p diminished autophagy and promoted the radiosensitivity of LUSC by negatively regulating LTF.

Moreover, as suggested by colony formation analysis, NEAT1 got in touch with promoting radioresistance in LUSC cells, while miR-214-5p inhibitors reversed NEAT1 knockdown-induced suppression of radioresistance (***Fig. S5C, D***). The mRFP-GFP-LC3 and western blot assay results also showed that the impact on autophagy of NEAT1 knockdown in H226R and H1703R cells could be reversed by miR-214-5p inhibitors (***Fig. 7C, D and Fig. S6C, D***). Consistently, *in vivo* experimental results demonstrated that knockdown of NEAT1 enhanced radiation-induced growth suppression of LUSC xenografts, while miR-214-5p inhibitors reversed this effect (***Fig. S7A, B***). Therefore, our findings disclosed that the NEAT1/miR-214-5p/LTF axis could promote autophagy, thereby increasing the radioresistance of LUSC cells.

### SP2 cooperated with HDAC1 to promote NEAT1 transactivation in LUSC cells

**Figure 8:**
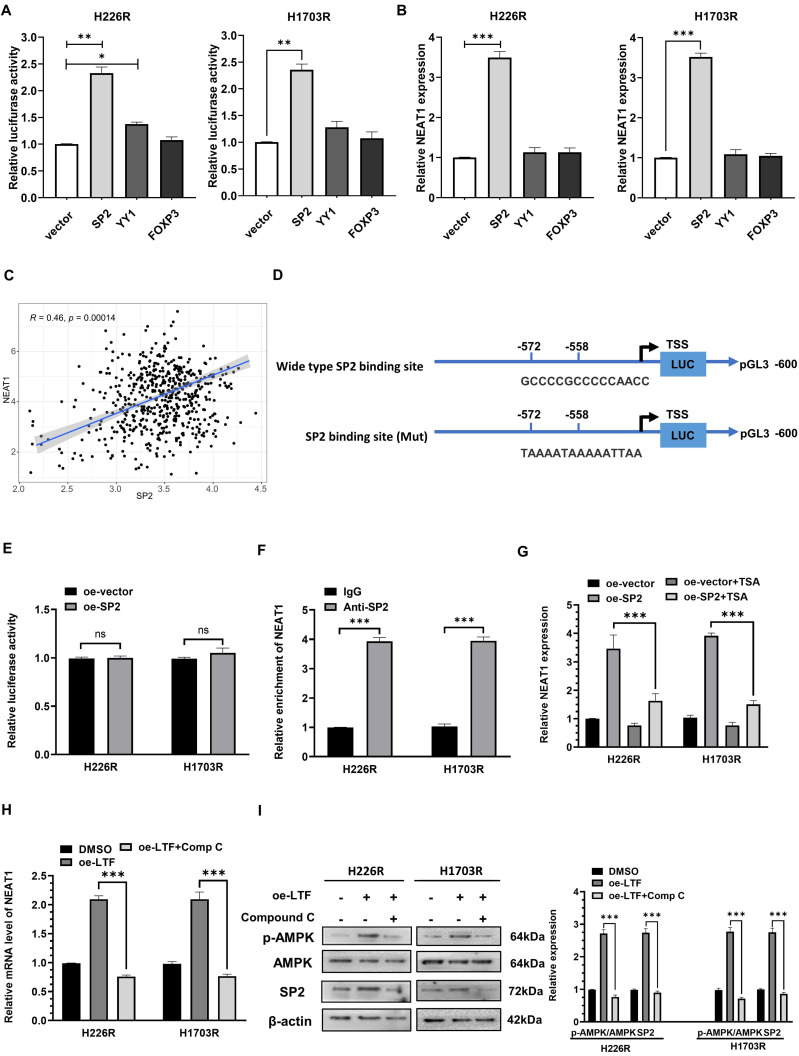
** SP2 promote NEAT1 transactivation by cooperating with HDAC1 in LUSC cells. (A)** Relative NEAT1 promoter activity in SP2-, YY1- and FOXP3-overexpressing H226R and H1703R cells. **(B)** Relative expression of NEAT1 in SP2-, YY1- and FOXP3-overexpressing H226R and H1703R cells.** (C)** Correlation between the expression level of SP2 and NEAT1 in TCGA LUSC cohort. **(D)** Wild type and mutant SP2 binding site on the NEAT1 promoter. **(E)** Relative activity of mutant NEAT1 promoter in SP2-overexpressing H226R and H1703R. **(F)** ChIP-qPCR analysis of SP2 occupancy on the promoter of NEAT1 in H226R and H1703R. **(G)** qRT-PCR analysis of NEAT1 expression in SP2-overexpressing H226R and H1703R cells in the presence of TSA (300 nM). **(H)** qRT-PCR analysis of NEAT1 in LTF-overexpressing H226R and H1703R cells treated with Comp C (10 μM). **(I)** H226R and H1703R cells were transfected with oe-LTF and then treated with Comp C (10 μM) and the expression of p-AMPK, AMPK and SP2 was assessed by western blotting. Error bars represent the mean ± SD of three independent experiments. *p < 0.05, **p < 0.01, ***p < 0.001. *p* < 0.05 was considered significant.

To explore the upstream controller of NEAT1, we predicted the transcription factors involved in NEAT1 transcription through JASPAR, Human Transcription Factor Database and PROMO database. SP2, YY1 and FOXP3 were screened out to be potential transcriptional regulators. Dual-luciferase reporter assays revealed that overexpression of SP2 or YY1 significantly increased NEAT1 promoter activity (***Fig. 8A***). However, only SP2 overexpression increased NEAT1 expression (***Fig. 8B***). Pearson correlation analysis of TCGA data indicated that NEAT1 expression was positively associated with SP2 expression in LUSC (***Fig. 8C***). These results suggested that SP2 positively regulated NEAT1 expression at the transcription level.

We then analyzed the potential SP2-binding site through the JASPAR website and generated NEAT1 promoter containing mutant predicted binding site at -572 to -558 bp upstream of the TSS (***Fig. 8D***). Mutation of the binding site abolished SP2-mediated induction of the NEAT1 promoter reporter activity, indicating that SP2 directly regulated the transcription of NEAT1 through binding with -572 to -558 bp region upstream of the TSS (***Fig. 8E***). In addition, ChIP-qPCR revealed that SP2 was remarkably enriched on the binding site of the NEAT1 promoter (***Fig. 8F***). These results indicated that SP2 binds to the promotor of NEAT1 and activates its transcription.

Previous research has shown that the transcriptional facilitated ability of SP2 requires the assistance of HDAC1 20,21, so we added trichostatin A (TSA), a repressor of HDAC 22,23, to determine whether it affected the promoted effect of SP2 on NEAT1. qRT-PCR results showed that SP2 lost its ability to induce NEAT1 transcription in the presence of TSA (***Fig. 8G***), revealing that SP2 enhanced NEAT1 expression via HDAC1-mediated transcriptional regulation. Moreover, we found that compound C (Comp C), an inhibitor of AMPK could reverse the upregulation of SP2 and NEAT1 induced by LTF overexpression (***Fig. 8H, I***). These data suggest that NEAT1 is transcriptionally upregulated by SP2 and functions as a radioresistant activator gene in LUSC via the LTF/AMPK/SP2/NEAT1/miR-214-5p feedback loop.

### Irradiation induced activation of the LTF/AMPK/SP2/NEAT1/miR-214-5p feedback loop in LUSC

**Figure 9:**
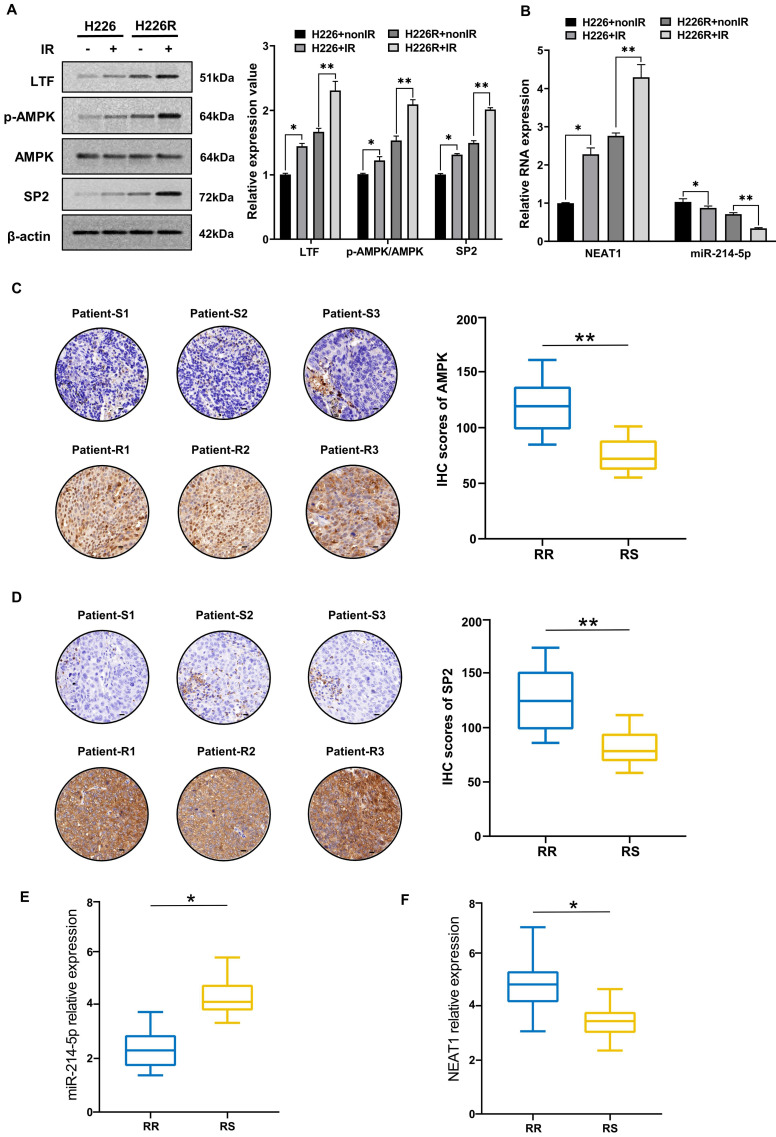
** Irradiation induced activation of the LTF/AMPK/SP2/NEAT1/miR-214-5p feedback loop in LUSC cells and tissues. (A)** Western blot assay of LTF, AMPK and SP2 proteins and relative expression levels in H226 and H226R cells with or without irradiation. **(B)** qRT- PCR analysis of NEAT1 and miR-214-5p in H226 and H226R cells with or without irradiation. **(C, D)** Representative immunohistochemistry staining against AMPK and SP2 in radiosensitive (patient-S1,2,3) and radioresistant (patient-R1,2,3) clinical LUSC samples (40X). Scale bars, 20 μm. **(E, F)** RT-qPCR analysis of NEAT1 and miR-214-5p expression in 10 LUSC radioresistant and 10 radiosensitive tissues. * p < 0.05 between indicated groups, ** p < 0.01 between indicated groups.

Next, we evaluated the contribution of irradiation in regulating the LTF/AMPK/SP2/NEAT1/miR-214-5p feedback loop in LUSC cell lines and clinical samples. We found that the expression of LTF, AMPK and SP2 in H226 and H226R cells were increased in response to irradiation **(*Fig. 9A*)**. The results of qRT-PCR also indicated that after exposure to a radiation dose of 6 Gy, the expression of NEAT1 increased and miR-214-5p decreased significantly **(*Fig. 9B*)**. Interestingly, marked changes in essential components of the LTF-signaling loop are noted in H226R cells, suggesting that irradiation favors enhancing the loop in radioresistant rather the counterpart radiosensitive LUSC cells. Same results can also be observed in H1703 and H1703R cells (***Fig. S8*)**. Meanwhile, immunohistochemistry showed that AMPK and SP2 proteins were significantly higher in the radioresistant clinical samples than the radiosensitive ones **(*Fig. 9C, D*)**. We also found that compare to the radiosensitive tissues, the levels of NEAT1 were upregulated while miR-214-5p were downregulated in LUSC radioresistant tissues **(*Fig. 9E, F*)**. In summary, our results demonstrated that irradiation could activate the LTF-signaling feedback loop in LUSC, and this effect was more pronounced in radioresistant tumors.

## Discussion

**Figure 10:**
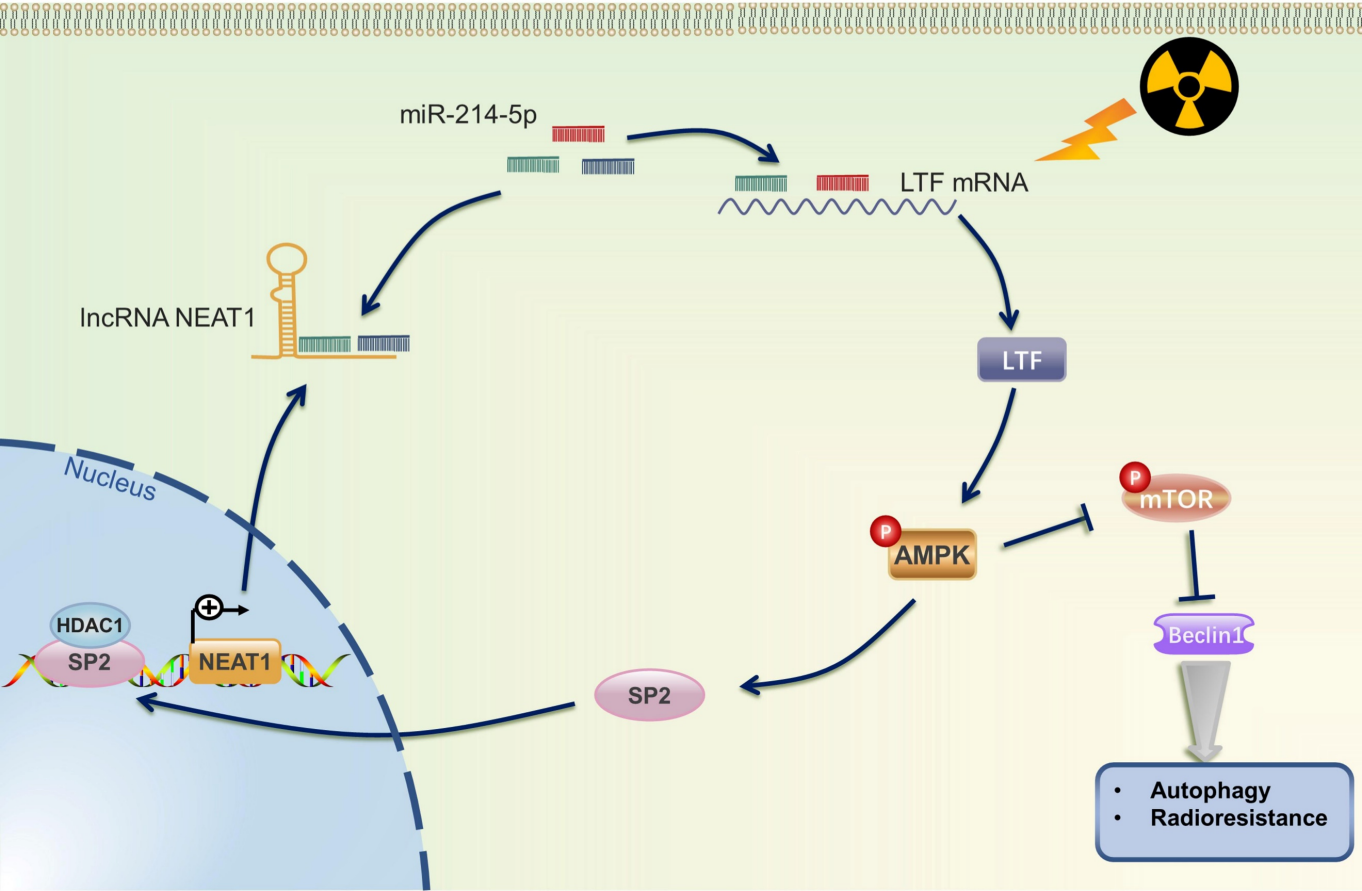
** The mechanistic scheme of this study.** Irradiation (IR) increased the expression of LTF, which, in turn, activated the AMPK signalling pathway and induced the autophagy signaling, further promoting radioresistance in LUSC cells. SP2, a downstream protein of AMPK, binding to the promoter region and promoted NEAT1 expression. Eventually, NEAT1 increased mRNA level of LTF through a ceRNA network by sponging miR-214-5p.

Radiotherapy, alone or combined with chemotherapy or ICIs, plays a vital role in the treatment of NSCLC. Howbeit, recurrence and distant metastasis after radiotherapy remain the major hindrance to improve long-term prognosis, and the molecular mechanism of LUSC radioresistance is still unsettled. In the present study, we first lay the foundation to show that LTF plays a radioresistance-promoting role in LUSC by activating the AMPK/mTOR signaling pathway and inducing autophagy. We also elucidated a complex molecular feedback circuit involving LTF, AMPK, SP2, and NEAT1/miR-214-5p in LUSC cells (***Fig. 10)***.

It has long been understood that autophagy can either induce type-II programmed cell death through the degradation of vital components or protect the cellular survival via adaptive response, thus, acting as a double-edged sword in regulating cancer cell survival 24,25. Our previous studies demonstrated that autophagy is one of the major modulators contributing to the radioresistance of malignant tumors 6,8,26, and investigating the mechanism of autophagy after irradiation is important for understanding radioresistance and for designing therapeutic approaches to overcome radioresistance and improving the efficiency of radiotherapy for lung cancer patients. Karagounis et.al found that repression of the autophagic function in lung cancer cells results in increased radiosensitivity 27. Keta et.al also indicated that inhibition of cytoprotective autophagy improved cytotoxicity induced by radiation on radioresistant lung adenocarcinoma cells 28. Consistent with these findings, our results also confirmed that autophagy makes LUSC more resistant to radiotherapy.

LTF, as indicated by the published studies, mainly functions as the regulator of innate immunity and nutrition 9,11. However, research conducted before showed that the expression of LTF is significantly heterogeneous in different tumors, suggesting its different roles in carcinogenesis 29. For instance, LTF is downregulated and acts as a tumor suppressor by repressing AKT signaling in nasopharyngeal carcinoma 13. In contrast, Qi et.al suggested that the down-regulated LTF enhances the radiosensitivity of nasopharyngeal carcinoma cells through interaction with miR-214 17. Moreover, its role in radioresistance in lung cancer has not yet been reported, thus, further investigation is warranted to fulfill this gap. At this point, after verifying the radioresistance ability of two radioresistant LUSC cell lines (H226R and H1703R), the RNA and proteomic analyses both revealed that LTF was the most significantly upregulated gene in radioresistant cancer cells. Previous studies have suggested that irradiation can upregulate the expression of proteins in cancer cells, especially the radioresistant cells 30. For example, PKP2 has been identified as a critical driver of radiation resistance in lung cancer, and its expression was significantly higher after irradiation than before irradiation 31. Lu also found that irradiation promoted mTOR expression and activation in pancreatic cancer cells through reducing miR-99b expression 32. We further found that silencing LTF expression not only reversed radioresistance but also inhibited autophagy in radioresistant LUSC cells. Further analysis revealed a potential signaling cascade of LTF in the regulation of autophagy: LTF directly interacts with AMPK and activates the AMPK/mTOR signaling. It is acknowledged that autophagy is regulated by a complex network; of these, the AMPK/mTOR pathway has been well characterized. Autophagy can be positively enhanced by AMPK activation and mTOR reduction. AMPK is known as an intracellular energy sensor and a mediator of autophagy, which promotes the radioresistance of various tumor cells 33,34. Our previous research and other studies have demonstrated that AMPK/mTOR-related autophagy makes a crucial contribution to the treatment resistance of tumor cells 6,35. In this study, silencing LTF expression decreased AMPK phosphorylation but increased mTOR phosphorylation in LUSC radioresistant cell lines, which suggested the AMPK/mTOR pathway plays an important role in LTF-induced autophagy activation and radioresistance in LUSC.

According to the ceRNA hypothesis, lncRNAs can competitively sponge miRNAs, inhibit the ability of miRNAs to induce the degradation of their target genes, and thus be involved in tumorigenesis and progression 36,37. In addition, recent studies have reported that lncRNAs, such as GAS5, TRPM2-AS, OIP5-AS1 act as ceRNAs and microRNA sponges to promote the expression of target genes and consequently enhance radioresistance in several cancers 38-40. LncRNA PVT1 may promote cell apoptosis through the miR-424-5p/PVT1/CARM1 signaling pathway, thereby enhancing the radiosensitivity of lung cancer cells 41. Yu et al. also found that inhibition of SBF2-AS1 expression may enhance the radiosensitivity and apoptosis of NSCLC through the SBF2-AS1/miR-302a/MBNL3 axis 42. Herein, by using bioinformatic and luciferase reporter assays, we explored the upstream regulatory mechanisms of LTF expression. miR-214-5p mimic reduced LTF mRNA and protein expressions in LUSC cells; however, LTF expression in the oe-NEAT1+miR-214-5p mimic group was preserved, indicating that NEAT1 antagonized the inhibitory effect of miR-214-5p on LTF expression. In contrast, reduced NEAT1 expression reversed the promotion of LTF expression induced by the miR-214-5p inhibitor. These results indicate that NEAT1 may increase the mRNA level of LTF through a ceRNA network by sponging miR-214-5p and promoting autophagy after irradiation, thereby inducing the radioresistance in LUSC.

SP2 is a renowned transcription factor, over-expressed in several human cancers, and implicated in an ample variety of essentially biological processes including cell growth, differentiation, apoptosis and carcinogenesis 20. Several lines of evidence indicated that HDAC1 is essential for the SP2 transcription activity 21. Intriguingly, our results revealed that SP2 binds the promoter region of NEAT1 by dint of HDAC1 and promotes its transcription. Moreover, NEAT1-induced LTF/AMPK activation may have increased SP2 expression, which in turn enhanced its effect on NEAT1 promoter transcription.

## Conclusions

To our knowledge, our study represents the first demonstration that after irradiation, LTF activates the AMPK pathway and enhances its self-expression via forming a LTF/AMPK/SP2/NEAT1/miR-214-5p positive-feedback loop to promote autophagy signaling and finally induce radioresistance in LUSC. Evidently, further investigation is warranted to explore whether it can be targeted in clinical settings as a novel radiosensitizer of lung cancer.

## Supplementary Material

Supplementary figures and tables.

## Abbreviations

LUSC: lung squamous cell carcinoma
NSCLC: non-small cell lung cancer
NC: negative control
OS: overall survival
RFS: recurrence-free survival
siRNA: small interfering RNA
TMT: tandem mass tag
TCGA: the Cancer Genome Atlas
LncRNAs: long noncoding RNAs
IHC: immunohistochemistry
qRT-PCR: quantitative Real-time PCR
ceRNA: competing endogenous RNA
