# Supplementary material for: LTF Induces Radioresistance by Promoting Autophagy and Forms an AMPK/SP2/NEAT1/miR-214-5p Feedback Loop in Lung Squamous Cell Carcinoma
Source: Int J Biol Sci. 2023 Feb 27;19(5):1509–27. doi: 10.7150/ijbs.78669 (PMC10086751; doi:10.7150/ijbs.78669)
Supplement: Supplementary file 1 — Supplementary figures and tables. [file ijbsv19p1509s1.pdf]

Additional Files

Supplementary Tables

Table S1. Primers for qPCR.

RNA	Sequences of primers
LTF	forward 5'-AGTCTACGGGACCGAAAGACA-3';<br>reverse 5'-CAGACCTTGCAGTTCGTTCAG-3'
lncRNA NEAT1	forward 5'-ATGCCACAACGCAGATTGAT-3';<br>reverse 5'-CGAGAAACGCACAAGAAGG-3'
miR-214-5p	forward 5'-ACACTCCAGCTGGGACAGCAGGCCAGAC-3';
β-actin	forward 5'-CATGTACGTTGCTATCCAGGC-3';<br>reverse 5'-CTCCTTAATGTCACGCACGAT-3'

Table S2. Sequences for the siRNA.

siRNA	Sequences
si-LTF-1	5'-CGGUGCAGAUAAAGGACAGUU-3'
si-LTF-2	5'-CCCUACAAACUGCGACCUGUA-3'
si-NEAT1	5′-GATCCGGGTTGGTTAGAGATA<br>CAGTGCTTCCTGTCAGACACTGTATCTCTAACCAACCCTTTTTG-3′
si-NC	5'-UUCUCCGAACGUGUCACGUTT-3'

Table S3. Antibodies used for western blot, RIP, ChIP and IHC.

Antibody	Catalog	Dilution	Company	Detection
LTF	10933-1-AP	1:1000	Proteintech	WB
P62	16177	1:1000	CST	WB
LC3	12741	1:1000	CST	WB
β-actin	3700	1:20000	CST	WB
LTF	ab109216	1:200	Abcam	IF
LTF	ab15811	1:100	Abcam	IHC
AMPK	ab32047	1:1000	Abcam	WB
AMPK	ab32047	1:250	Abcam	IF
mTOR	ab109268	1:1000	Abcam	WB
Beclin1	ab207612	1:2000	Abcam	WB
c-MYC	ab9106	1:1800	Abcam	Co-IP
FLAG	ab49763	1:1000	Abcam	Co-IP
SP2	25000-1-AP	1:1000	Proteintech	WB
HRP conjugated goat anti-rabbit IgG	AP106P	1:5000	Sigma-Aldrich	WB

HRP conjugated goat anti-mouse IgG	AP127P	1:5000	Sigma-Aldrich	WB

Table S4. Correlation between LTF expression and clinico-pathologic characteristics of LUSC.

**Variables**		**LTF expression**		**p-value**
		**High (n = 56)**	**Low (n = 54)**	
Age (mean (SD))		63.43 (9.77)	63.12 (10.01)	0.541
Gender (%)	Female	26 (46.4)	23 (42.6)	0.164
	Male	30 (53.6)	31 (57.4)	
Smoking (%)	Never	18 (47.4)	14 (25.9)	0.972
	Ever	38 (52.6)	30 (74.1)	
Histological grade	Well/Moderate	12 (21.4)	43 (79.6)	<0.001
	Poor	44 (78.6)	11 (20.4)	
8th TNM stage (%)	I-II	12 (21.4)	35 (64.8)	<0.001
	III-IVA	44 (78.6)	19 (35.2)	
Tumor Location (%)	Left	27 (48.2)	26 (48.1)	0.994
	Right	29 (51.8)	28 (51.9)	

Supplementary Figures

Fig. S1

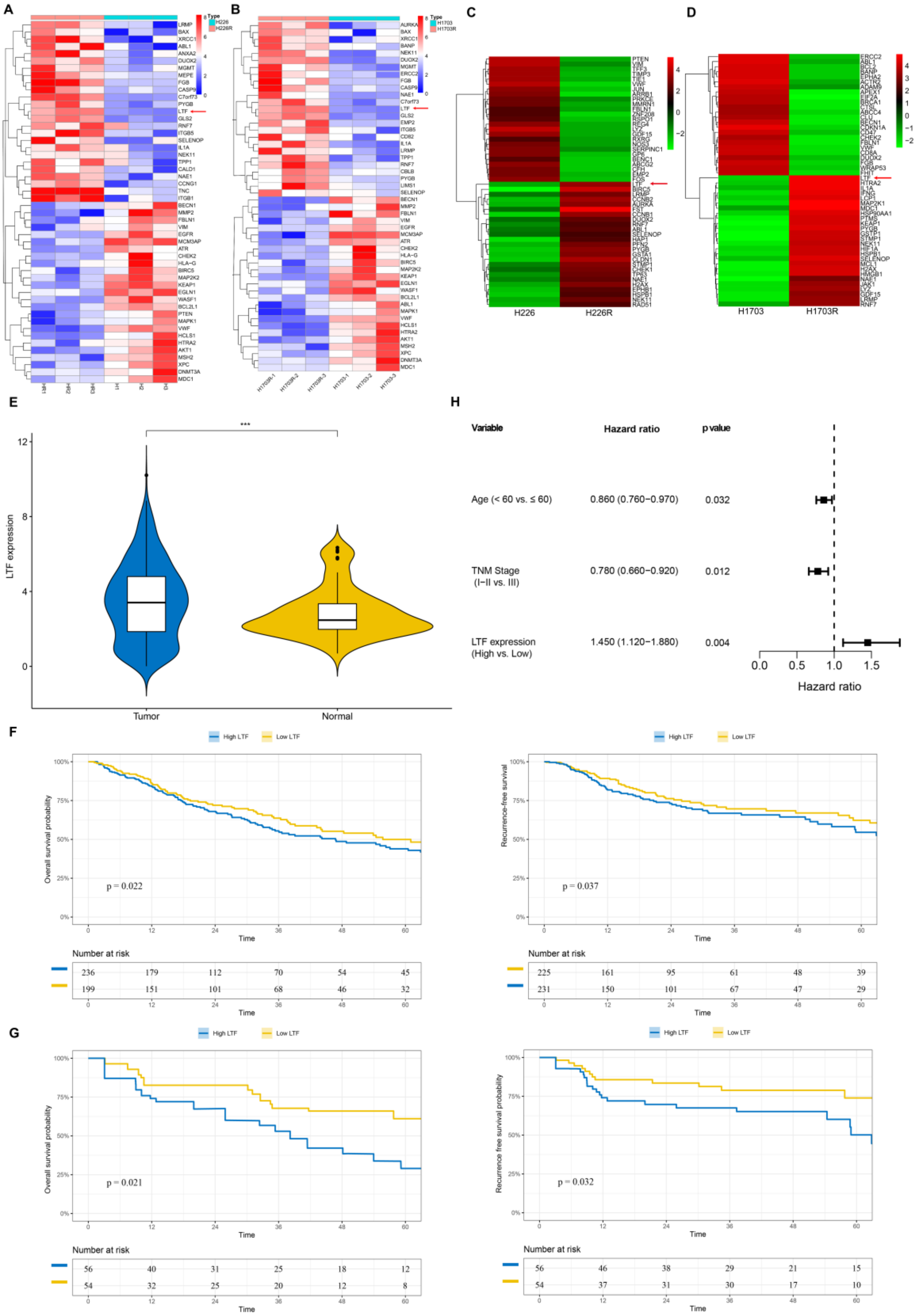

Fig. S1. Levels of LTF were clearly upregulated and correlated with

radioresistance and prognosis in LUSC tissues and LUSC cell lines. **(A)** Clustered heatmap of RNA-seq data showing the top 50 differentially expressed (up- and downregulated) genes in H226R and H226 cells. The red arrowhead shows the LTF gene. **(B)** Clustered heatmap of RNA-seq data showing the top 50 differentially expressed (up- and downregulated) genes in H1703R and H1703 cells. **(C)** Heat maps of the top 50 differentially expressed proteins from mass spectrometry between H226R and H226 cells. Red arrowhead indicates the LTF gene. **(D)** Heat maps of the top 50 differentially expressed proteins from mass spectrometry between H1703R and H1703 cells. Red arrowhead indicates the LTF gene. **(E)** TCGA analysis of LTF expression in LUSC compared with adjacent normal tissues. The data are presented as the mean ± SD values. ***$p < 0.001$. **(F, G)** Survival analysis of LUSC patients underwent RT from TCGA cohort (F) and the FUSCC cohort (G) in the LTF-high and LTF-low groups. **(H)** Multivariate Cox regression analysis of LTF.

Fig. S2

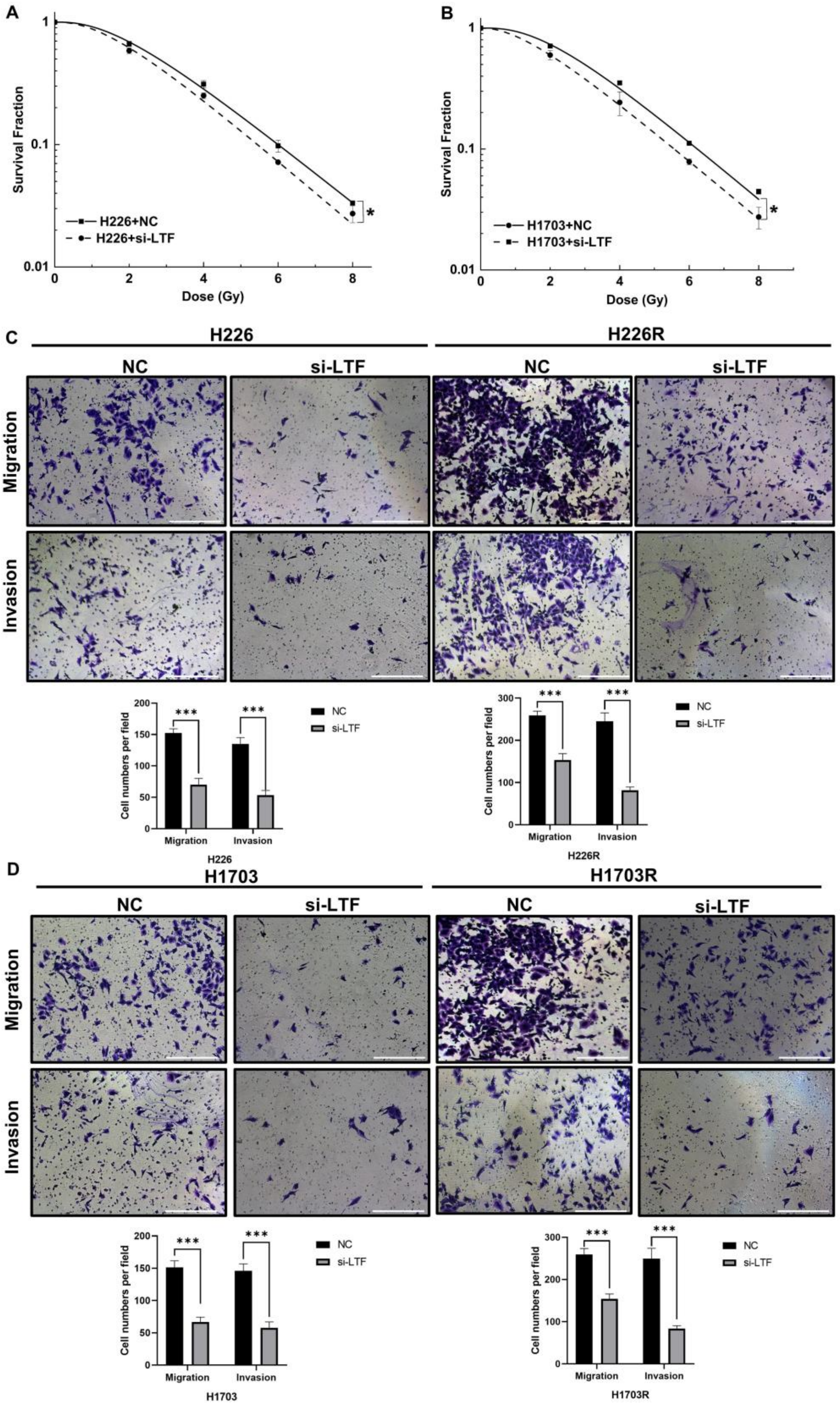

Fig. S2. Relationship of LTF expression with radiosensitivity of LUSC

cells. (A, B) Dose responses of survival factions of H226 and H1703 cells before and after si-LTF transfection. **$p < 0.01$, ***$p < 0.001$ between indicated groups. **(C)** The cell migration and invasion ability evaluated by in vitro transwell assay after LTF silence in H226 and H226R cells. ***$p < 0.001$ between indicated groups. **(D)** The cell migration and invasion ability evaluated by in vitro transwell assay after LTF silence in H1703 and H1703R cells. **$p < 0.01$, ***$p < 0.001$ between indicated groups. Scale bars, 100 μm.

Fig. S3

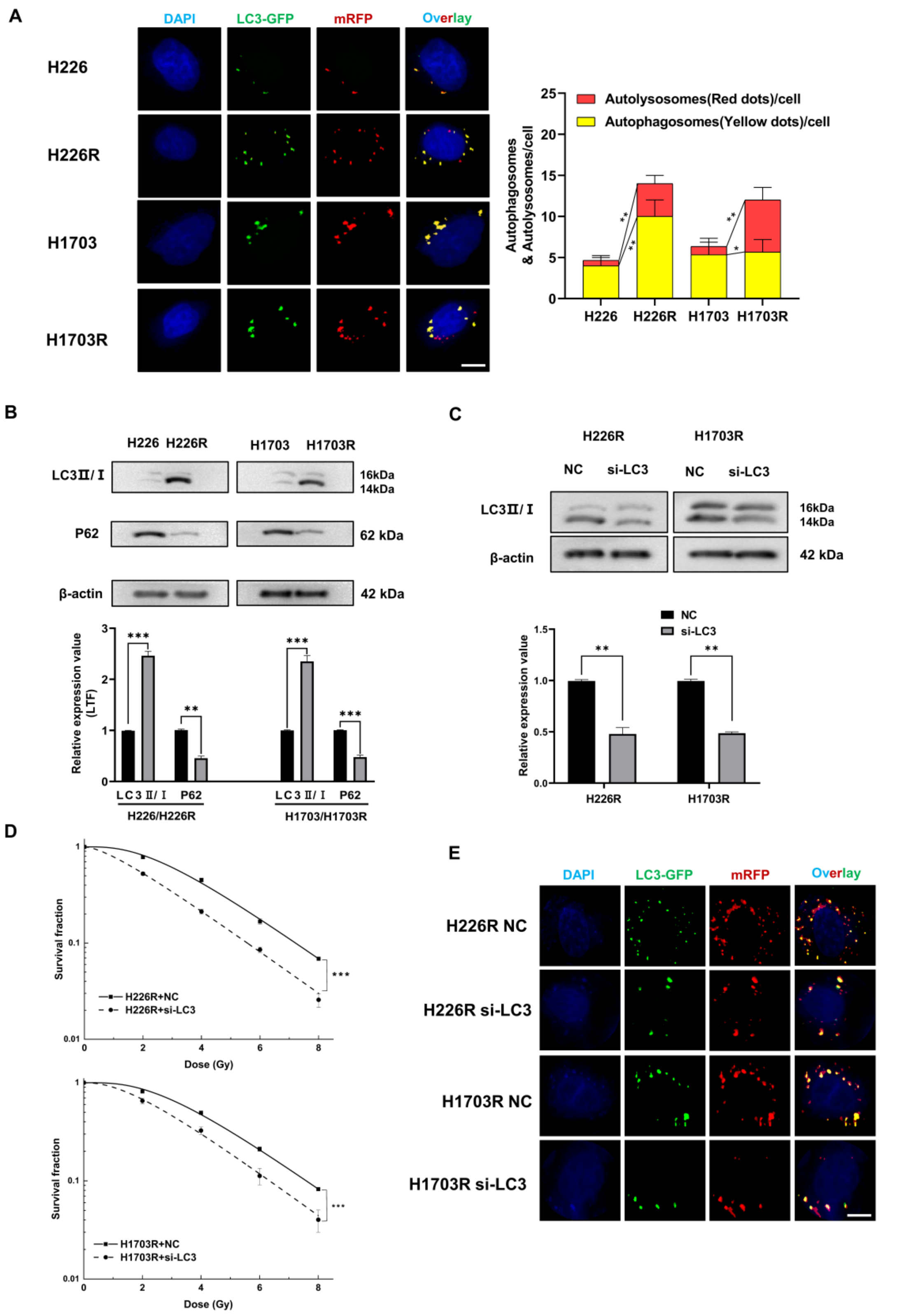

Fig. S3. Depletion of autophagy contributes to radiosensitization of LUSC cells. (A) Fluorescence images of H226, H226R, H1703 and

H1703R cells transfected with mRFP-GFP-LC3-tagged adenovirus (×40). Red dots indicate autolysosomes while yellow dots indicate autophagosomes in overlays. Nuclei were stained with DAPI. Scale bars: 10 μm. The average number of autophagosomes and autolysosomes in each indicated cell was quantified. $**p < 0.01$. **(B)** Western blot assay of P62 and LC3 proteins in H226, H226R, H1703 and H1703R cells. $**p < 0.01$ between indicated groups. **(C)** Efficiency of siLC3 transfection in H226R and H1703R cells. $**p < 0.01$ between indicated groups. **(D)** Dose responses of survival factions of H226R (left) and H1703R (right) cells before and after si-LC3 transfection. $***p < 0.001$ between indicated groups. **(E)** Fluorescence images siLC3-interfered H226R and H1703R cells transfected with mRFP-GFP-LC3-tagged adenovirus (×40).

Fig. S4

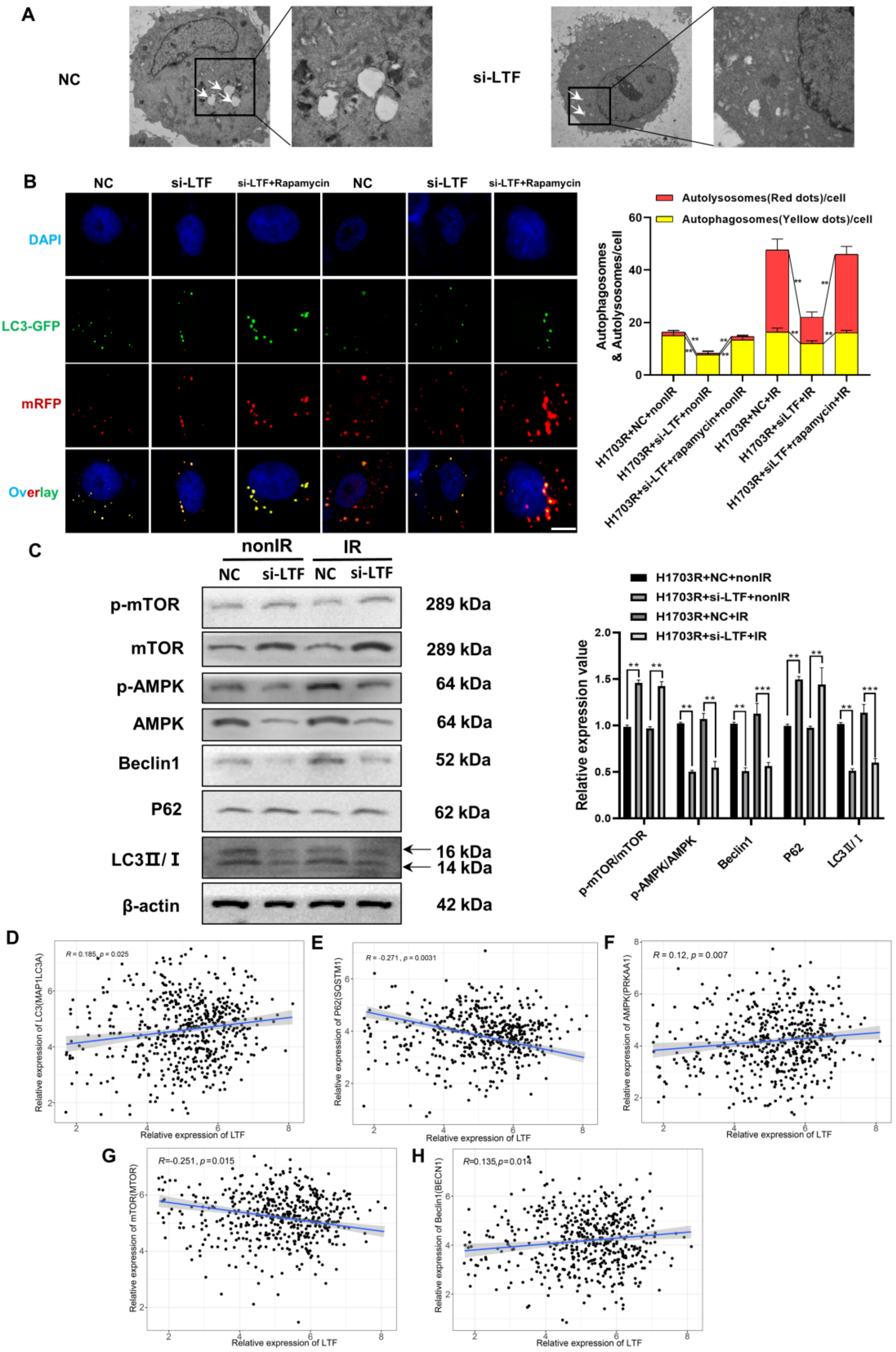

Fig. S4. LTF knockdown by siRNA inhibited autophagy via the AMPK/mTOR/Beclin1 axis in LUSC cells. (A) The autophagosomes of

H1703R cells with control or stable knockdown of LTF were examined with transmission electron microscopy (TEM). Autophagosomes was indicated by white arrows. Scale bar = 5 μm (left) or Scale bar = 1 μm (right). **(B)** Fluorescence images of H1703R cells transfected with si-LTF and mRFP-GFP-LC3-tagged adenovirus (×40) after 6 Gy irradiation or not. Red dots indicate autolysosomes while yellow dots indicate autophagosomes in overlays. Nuclei were stained with DAPI. Scale bars: 10 μm. The average number of autophagosomes and autolysosomes in each indicated cell was quantified. **p < 0.01. **(C)** Western blot analysis of mTOR, p-mTOR, AMPK, p-AMPK, Beclin1, P62 and LC3 expression in nonirradiated or irradiated H1703R cells following si-LTF transfection. ** $p < 0.01$ between indicated groups, *** $p < 0.001$ between indicated groups. **(D-H)** The association of LTF and LC3 (D), P62 (E), AMPK (F), mTOR (G) as well as Beclin1 (H) in LUSC patients using the FUSCC datasets (Correlation was assessed using Spearman correlation coefficient).

Fig. S5

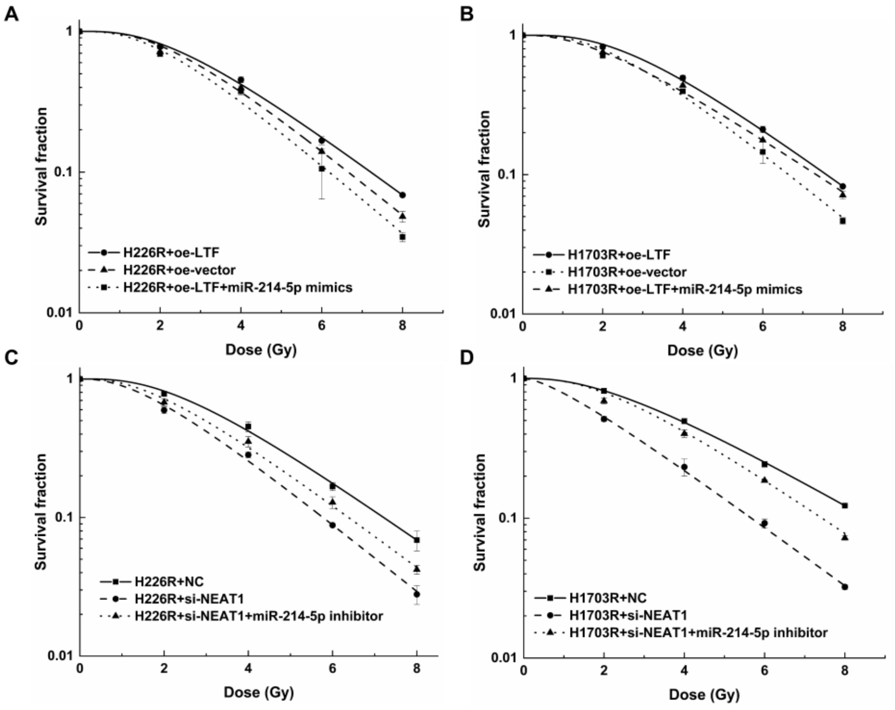

Fig. S5. The NEAT1/miR-214-5p/LTF axis is involved in autophagy-mediated radioresistance of LUSC cells. Clonogenic survivals showed miR-214-5p mimics reversed the radioresistance caused by the overexpression of LTF in H226R **(A)** and H1703R **(B)** cells. Clone formation assays were applied to detect the survival fraction of H226R **(C)** and H1703R **(D)** cells transfected with si-NEAT1, si-NEAT1 plus miR-214-5p inhibitor, or the control after X-ray irradiation.

Fig. S6

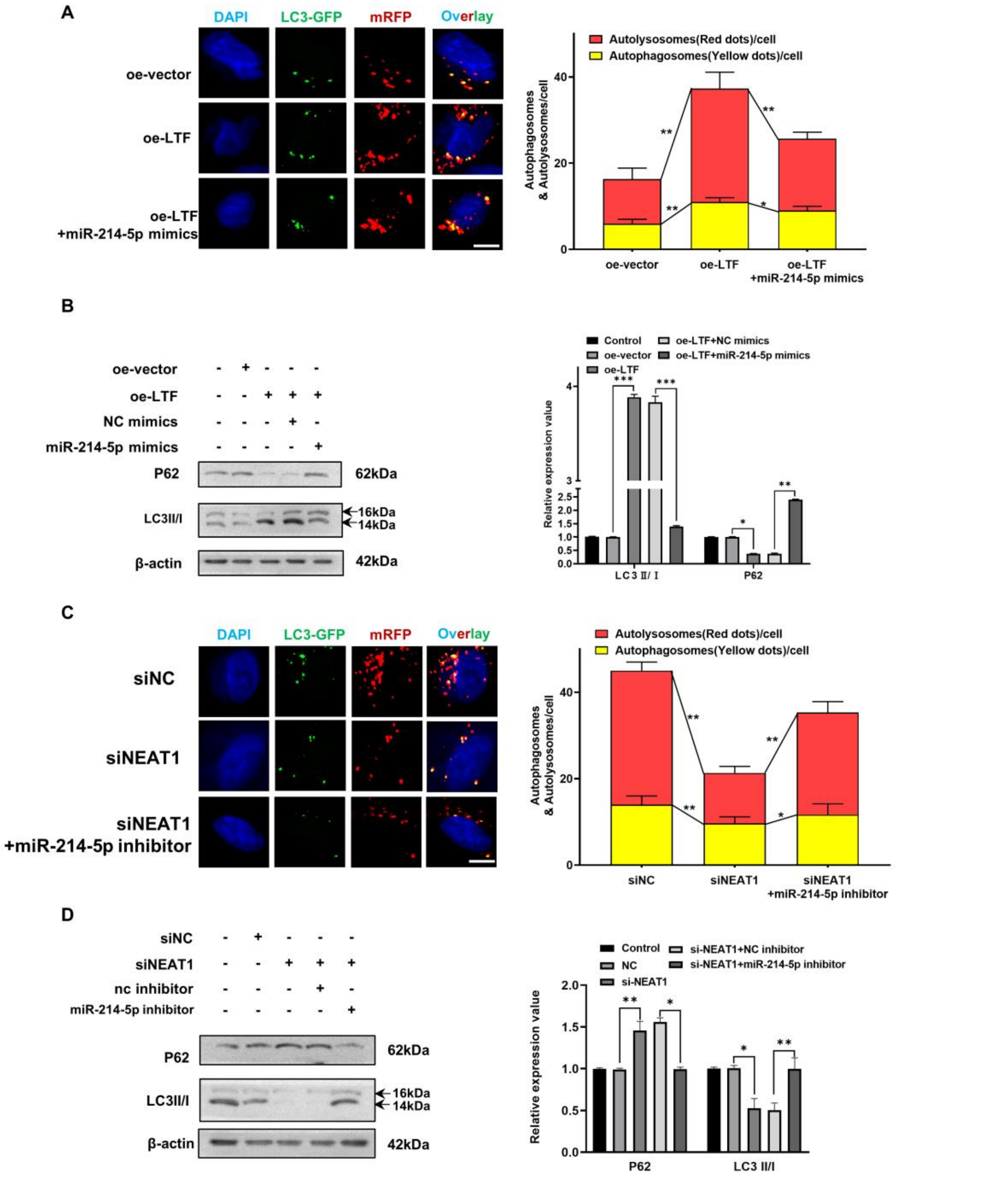

Fig. S6. The NEAT1/miR-214-5p/LTF axis is involved in autophagy-mediated radioresistance of H1703 cells. (A) mRFP-GFP-LC3 assay showed miR-214-5p mimics reversed the effects of oe-LTF on autophagy in H1703R cells. Red dots indicate autolysosomes while yellow dots indicate autophagosomes in overlays. Nuclei were stained with DAPI.

Scale bars: 10 μm. The average number of autophagosomes and autolysosomes in each indicated cell was quantified. **$p < 0.01$. **(B)** Western blot assay showed miR-214-5p mimics reversed the effects of oe-LTF on autophagy in H1703R cells. **(C)** The effect on H1703R cell autophagy levels following transfected with si-NEAT1, si-NEAT1 plus miR-214-5p inhibitor, or the control after X-ray irradiation is tested by autophagic flux analysis. **(D)** Western blots identified the autophagy-related protein expression changes in si-NEAT1 and si-NEAT1 plus miR-214-5p inhibitor transfected H1703R cells. β-actin was used as a control.

Fig. S7

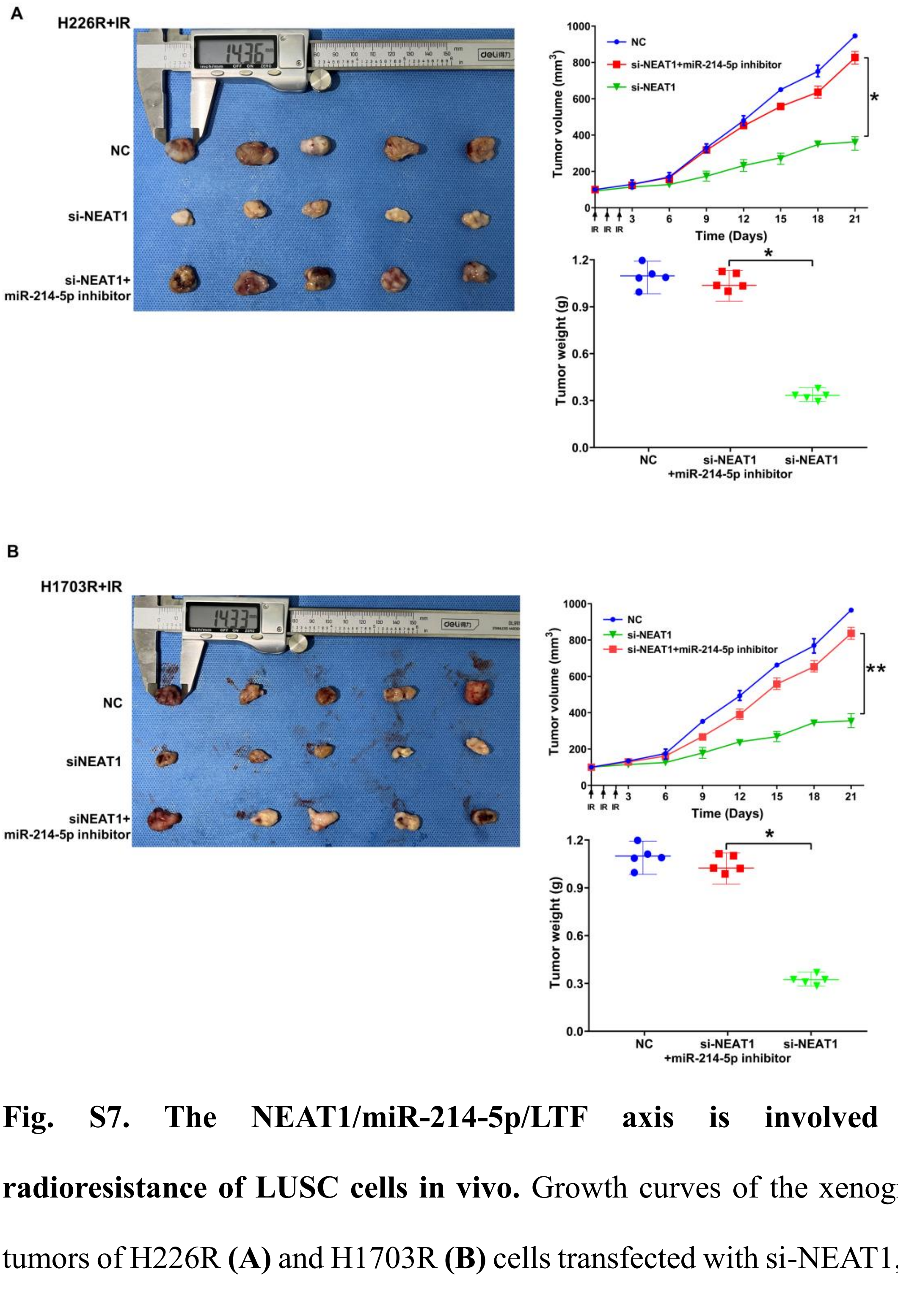

Fig. S7. The NEAT1/miR-214-5p/LTF axis is involved in radioresistance of LUSC cells in vivo. Growth curves of the xenograft tumors of H226R **(A)** and H1703R **(B)** cells transfected with si-NEAT1, si-NEAT1 plus miR-214-5p inhibitor, or the control and underwent 3 × 8 Gy fractionated X-ray irradiation. Tumor volume was measured every three days with a digital caliper and calculated using the formula (L $\times W^2$) × π/6.

Images of the dissected tumors from athymic nude mice (n = 5) were also shown.

Fig. S8

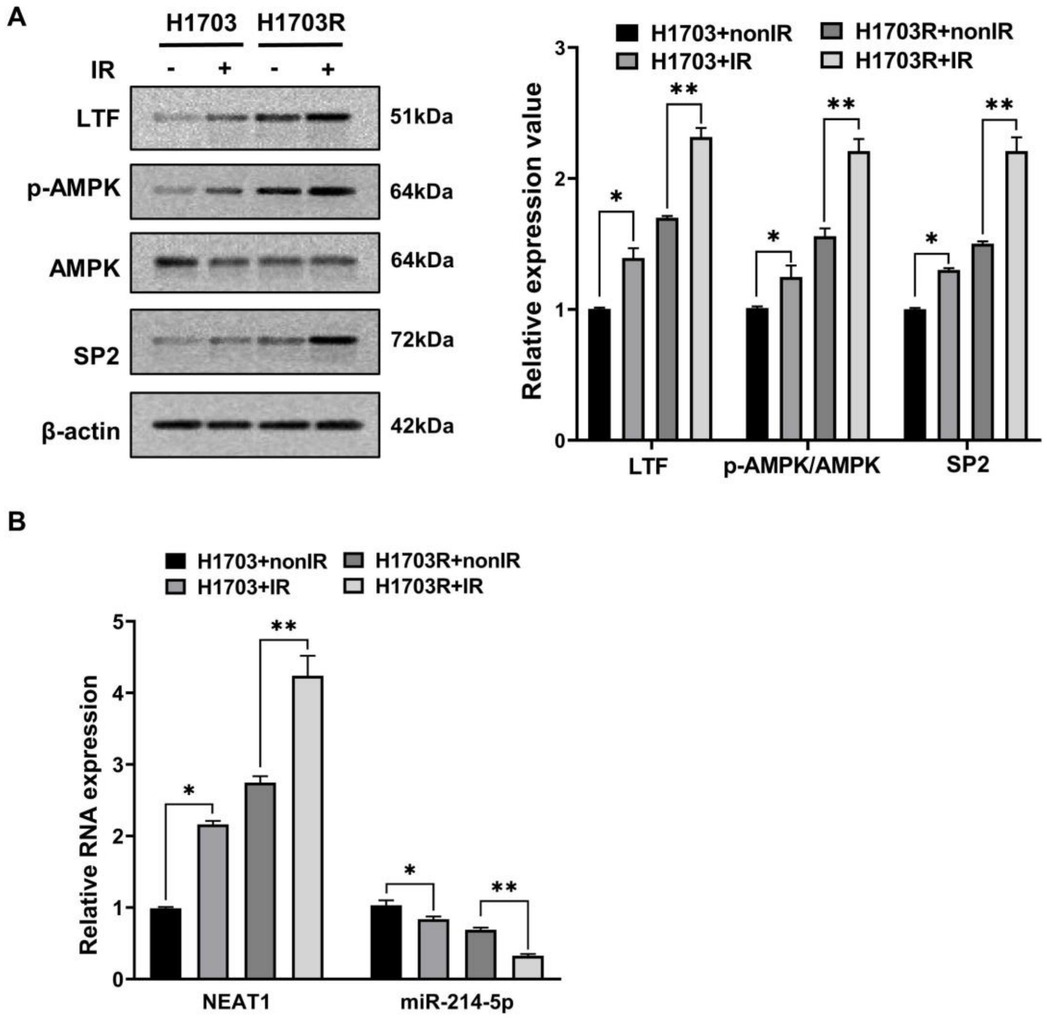

Fig.S8. Irradiation induced activation of the LTF/AMPK/SP2/NEAT1/miR-214-5p feedback loop in H1703 and H1703R cell lines. (A) Western blot assay of LTF, AMPK and SP2 proteins and relative expression levels in H1703 and H1703R cells with or without

irradiation. (B) qRT- PCR analysis of NEAT1 and miR-214-5p in H1703 and H1703R cells with or without irradiation. * $p < 0.05$ between indicated groups, ** $p < 0.01$ between indicated groups.
